# Consequences of somatic mutations of GIRK1 detected in primary malign tumors on expression and function of G-protein activated, inwardly rectifying, K^+^ channels

**DOI:** 10.3389/fonc.2022.998907

**Published:** 2022-10-31

**Authors:** Brigitte Pelzmann, Ahmed Hatab, Susanne Scheruebel, Sonja Langthaler, Theresa Rienmueller, Armin Sokolowski, Astrid Gorischek, Dieter Platzer, Klaus Zorn-Pauly, Stephan W. Jahn, Thomas Bauernhofer, Wolfgang Schreibmayer

**Affiliations:** ^1^Gottfried Schatz Research Center for Cell Signaling, Metabolism and Aging, Medical Physics and Biophysics, Medical University of Graz, Graz, Austria; ^2^Research Unit on Ion Channels and Cancer Biology, Medical University of Graz, Graz, Austria; ^3^Institute of Health Care Engineering with European Testing Center of Medical Devices, Graz University of Technology, Graz, Austria; ^4^Department of Dental Medicine and Oral Health, Medical University of Graz, Graz, Austria; ^5^Diagnostic & Research Institute of Pathology, Medical University of Graz, Graz, Austria; ^6^Division of Oncology, Department of Internal Medicine, Medical University of Graz, Graz, Austria

**Keywords:** ion channels and cancer, GIRK1, somatic mutation analysis, *Xenopus laevis* (*X. laevis*), confocal laser scanning microcopy, two electrode voltage clamp

## Abstract

A search in the GDC Data Portal revealed 304 documented somatic mutations of the KCNJ3 gene in primary tumors (out of 10.202 cases). Most affected tumor types were carcinomas from uterus, skin and lung, while breast cancer exerted the lowest number of somatic mutations. We focused our research on 15 missense mutations within the region between TM1 and TM2, comprising the pore helix and ion selectivity signature. Expression was measured by confocal laser scan microscopy of eGFP tagged GIRK1 subunits, expressed with and without GIRK4 in oocytes of *Xenopus laevis*. GIRK ion currents were activated *via* coexpressed m_2_Rs and measured by the Two Electrode Voltage Clamp technique. Magnitude of the total GIRK current, as well as the fraction of current inducible by the agonist, were measured. Ion selectivity was gauged by assessment of the P_Na+_/P_K+_ ratio, calculated by the GIRK current reversal potential in extracellular media at different Na^+^ and K^+^ concentrations. None of the tested mutations was able to form functional GIRK1 homooligomeric ion channels. One of the mutations, G145A, which locates directly to the ion selectivity signature, exerted an increased P_Na+_/P_K+_ ratio. Generally, the missense mutations studied can be categorized into three groups: (i) normal/reduced expression accompanied by reduced/absent function (S132Y, F136L, E139K, G145A, R149Q, R149P, G178D, S185Y, Q186R), (ii) normal/increased expression as well as increased function (E140M, A142T, M184I) and (iii) miniscule expression but increased function relative to expression levels (I151N, G158S). We conclude, that gain of function mutations, identical or similar to categories (ii) and (iii), may potentially be involved in genesis and progression of malignancies in tissues that exert a high rate of occurrence of somatic mutations of KCNJ3.

## Introduction

Chains of discrete genetic events, involving single mutations in the genome of cells of the body, give rise to the gradual transformation of a normal cell into a cancer cell. Accordingly, cancer can be well considered a genetic disorder of somatic cells. Random mutation is the driving force of this progressive transformation, hence genes that are exceptionally vulnerable are called ‘cancer driver’ genes. By a process analogous to Darwinian selection, clones with superior properties for proliferation and/or the successful passing over of anticancer defense mechanisms are sorted out, providing the basis for the next step in this vicious course, i.e., the progression of cancer (1). Besides a growing list of well-established cancer-driver genes (see e.g (2):), the contribution of genes encoding transmembrane ion-channels is increasingly recognized (3). Worthwhile to emphasize in this context are G-protein activated, inwardly rectifying, potassium channels (GIRKs). Following the binding of extracellular ligands to G-protein coupled receptors (GPCRs), membrane delimited pertussis toxin-sensitive G-protein βγ subunits are released and directly activate GIRK ion channels. Genes encoding GIRK subunits comprise KCNJ3 (Kir3.1), KCNJ6 (Kir3.2), KCNJ9 (Kir3.3) and KCNJ5 (Kir3.4). These subunits form heterotetrameric and/or homotetrameric ion channels in the plasma membrane (4). Important physiological and pathophysiological roles of GIRK channels have been discovered in the nervous system, heart, pancreas, blood platelets and in the regulation of lipid metabolism in fat cells and, as a consequence, GIRKs represent promising therapeutic targets (5–15). GIRK1 is virtually undetectable in healthy breast tissue. In contrast, it exhibits prominent neoplastic overexpression in a subset of estrogen receptor-positive (ER+) primary tumors of the breast (16), going hand in hand with increased lymph node metastasis and reduced survival of patients (17–19). Accordingly, moderate overexpression of GIRK1 mRNA has been observed in the MCF7 cell line that has been cultured from malignant breast tumour (20). Ectopic hyperexpression of GIRK1 has been shown to reinforce cancer hallmarks, such as cellular motility, invasiveness and angiogenesis in MCF7 cells (21). In contrast to MCF7, the MCF10A cell line was derived from benign breast epithelium and per se lacks detectable expression of GIRK1. Overexpression of GIRK1 in the benign MCF10A cell line resulted in an increase of cell motility, impairment of wound healing and extracellular matrix interactions and the induction of several cellular pathways towards pro-tumorigenic action as revealed by comparison of transcriptomes of original MCF10A cells with those ectopically overexpressing GIRK1 (22). While GIRK1 has been shown to play an important role in tumorigenesis and progression of breast tumors *via* irregular overexpression, *somatic mutations* in KCNJ5, the gene encoding the GIRK4 subunit, have been shown to be important driver mutations for producing sporadic primary aldosteronism, a benign alteration of the adrenal gland resulting in dysregulation of aldosterone production and resulting hypertension (23). Based on these facts we were interested to target the question whether somatic mutations of KCNJ3 have been already identified in malignant tumors and whether these mutations are functional and have effects on expression and function. The Xenopus laevis oocyte expression system, that has been used as a model system in the previous study, has been particularly useful to study the correlation between molecular structure, i.e. *in vitro* site directed mutagenesis and the electrophysiological, biochemical and cell biological function of receptors or channels in a standardized environment isolated from peculiar properties of a given cell type.

## Materials and methods

### Reagents and solutions

*ND96*: 96 mmol/L NaCl, 2 mmol/L KCl, 1 mmol/L MgCl_2_, 1 mmol/L CaCl_2_, 5 mmol/L Hepes, buffered with NaOH to pH 7.4; *NDE:* same as ND96, but CaCl_2_ was 1.8 mmol/L and 2.5 mmol/L pyruvate and 0.1% antibiotics (G-1397; ·1000 stock from Sigma-Aldrich) were added; *HK:* 96 mmol/L KCl, 2 mmol/L NaCl, 1 mmol/L MgCl_2_, 1 mmol/L CaCl_2_, 5 mmol/L Hepes buffered with NaOH to pH 7.4; *HK72, HK24, HK8, HK2 and HK0:* similar to HK, but K^+^ concentration was 72, 24, 8, 2 and 0 mmol/L and Na^+^ concentration was 26, 74, 90, 96 and 98 mmol/L, respectively. *GIRK1-Ab:* Anti-Kir3.1 (APC-005; Alomone Labs, Ltd, Jerusalem, Israel). *GAPDH-Ab:* Proteintech Cat.No.: 60004 (Proteintech; Am Klopferspitz 19, 82152, Planegg-Martinsried Germany). *KHA2 antisense oligonucleotide* was synthesized by Mycrosynth (“desalted” grade; Mycrosynth AG, CH-9436 Balgach, Switzerland). All other reagents used were of reagent grade throughout if not stated otherwise.

### Xenopus laevis oocyte expression, electrophysiology and confocal laser scan microscopy

The preparation of oocytes, two electrode voltage clamp technique (TEVC) and confocal microscopy (cLSM) on frog oocytes was performed as described previously (20). Briefly, approximately 24 h after their isolation, the oocytes were injected with the correct amount of mRNA and KHA2 antisense oligonucleotide, in order to knock down endogenous subunit (24). For a quantitative comparison of the level of expression and of the resulting GIRK currents (as shown in **Figures 2**, **3**, **4A**, **5A** and **Supplementary Tables 2-4**), the following amounts of mRNA and antisense oligonucleotide in ng per oocyte were injected: GIRK1^eGFP^ (WT as well as mutants): 1, GIRK4: 1, m_2_R: 1.5 and KHA2: 25 (KHA2 was always injected at the given amount, unless stated otherwise). Injected oocytes were kept in NDE at 19°C for 3–5 days before experiments were performed. For TEVC, oocytes were placed in a recording chamber, allowing superfusion with ND96, HK and HK with 10^-5^ mol/L acetylcholine at 21°C. Currents were recorded using agarose cushion electrodes (25) and the Geneclamp 500 amplifier (Axon Instruments, Foster City, CA, USA). Membrane potential was kept at -80 mV and the medium was changed from ND96 → HK → HK+acetylcholine following washout (HK+acetlycholine → ND96). Current traces were low pass filtered at 10 Hz and digitized at a sampling rate of 4 ms using the Digidata 1550B interface (Axon Instruments, Foster City, CA, USA) connected to Intel based computer running PClamp 11.0 software (Axon Instruments, Foster City, CA, USA). For oocytes that exerted currents > 10 μA in HK + acetylcholine, the medium was changed to HK24 + acetylcholine and the current amplitude was corrected empirically (multiplication factor for correction of I_HK24_ was 1.612 ± 0.079 (SEM), derived from 8 experimental days). Immediately after TEVC had been completed, the same oocytes were undertaken cLSM to quantify the amount of eGFP labeled GIRK1 subunit at or near the oocyte’s plasma membrane (20). The P_Na+_/P_K+_ ratio was assessed similar as described by (26). Briefly, G_β_ (2.5 ng/oocyte) and G_γ_ (0.5 ng/oocyte) subunit mRNA was injected in order to achieve permanent GIRK activation. When required, the amount of GIRK1 and GIRK4 was changed (in comparison to the experimental paradigm for the expression/function experiments), to yield robust ion currents in HK72, ranging from 1 to 4 μA, to make accurate assessment of the reversal potential possible. The amounts of mRNA injected for the different constructs were as follows (GIRK1^eGFP^/GIRK4 mRNA in ng/oocyte): GIRK4 (homooligomer): 5-10; WT/GIRK4: 0.65/0.65; S132Y/GIRK4: 1.25/1.25; L135I/GIRK4: 0.1-0.5/0.1-0.5; F136L/GIRK4: 0.5-5/0.5-5; E140M/GIRK4: 2.5/2.5; A142T/GIRK4: 0.1/0.1; G145A/GIRK4: 0.5-2.5/0.5-2.5; R149Q/GIRK4: 1.25-12.5/1.25-12.5; I151N/GIRK4: 12.5/12.5; M184I/GIRK4: 0.2-0.5/0.2-0.5 and Q186R/GIRK4: 1/1. After incubation the oocytes were subjected to TEVC. Membrane potential was kept at -80 mV and after the holding current had attained a stable value, perfusion was changed and K^+^: concentration increased stepwise from ND96 → HK2 → HK8 → HK24 → HK72. The entire experiment was recorded continuously at a sampling rate of 4 ms, using a Minidigi 1A digitizer (Axon Instruments, Foster City, CA, USA). After the holding current had achieved a stable value at a given perfusion medium, a voltage ramp with a duration of 2s from -120 mV to +50 mV was applied and current as well as voltage were recorded separately (sample rate 2 ms; Digidata 1550B; Axon Instruments, Foster City, CA, USA). Subsequently, the perfusion media were changed in the reverse order, but 2 mmol/L Ba^2+^ were present in the media, in order to block currents through GIRK channels (see **Figure 6A** for experimental protocol). The background ramp current recorded under 2 mmol/L Ba^2+^ for a given medium was subtracted from the ramp current measured without Ba^2+^ and plotted as a function of the measured membrane potential and the reversal potential was measured (see **Figures 6B, C**). For a given pair of reversal potentials ( *E*_*rev*1_ and*E*_*rev*2_ ), measured at two different extracellular K^+^ and Na^+^ concentrations ( ${\left[K^{+}\right]}_{1}^{0}$ $K^{+}{]}_{2}^{0}$ ${\left[Na^{+}\right]}_{1}^{0}$ and $Na^{+}{]}_{2}^{0}$ respectively), the Goldmann equation was solved for the permeability ratio $\frac{P_{Na^{+}}}{P_{K^{+}}}$ (Eqns. (1-3)):

Eqn. (1)


$$\frac{P_{Na^{+}}}{P_{K^{+}}}=\frac{{\left[K^{+}\right]}_{1}^{0}-A\cdot {\left[K^{+}\right]}_{2}^{0}}{A.{\left[Na^{+}\right]}_{2}^{0}-{\left[Na^{+}\right]}_{1}^{0}}$$

where *A* is,

Eqn. (2)


$$A=\frac{e^{\frac{E_{rev1}}{v_{0}}}}{e^{\frac{E_{rev2}}{v_{0}}}}$$

and *v_0_* is,

Eqn. (3)


$$v_{0}=\frac{R.T}{z.F}$$

Subsequently, $\frac{P_{Na^{+}}}{P_{K^{+}}}$ was calculated from *E*_*rev*1_ And *E*_*rev*2_ and the predetermined concentrations ${\left[K^{+}\right]}_{1}^{0}$ ${\left[K^{+}\right]}_{2}^{0}$ ${\left[Na^{+}\right]}_{1}^{0}$ and ${\left[Na^{+}\right]}_{2}^{0}$ . In equation (3), *R* represents the gas constant, *T* the absolute temperature, *z* the valence (i.e. 1 for Na^+^ and K^+^) and *F* the Faraday constant. For constructs that did not yield currents that were big enough to allow the exact determination of the P_Na+_/P_K+_ ratio (i.e.: E139K, R149P, G158S, G178D and S185Y), the maximum amount of mRNA encoding GIRK1 and GIRK4 possible (i.e. 12.5 ng/oocyte per mRNA species) along with m_2_R (1.5 ng/oocyte) was injected. These oocytes were undertaken a simplified, entirely qualitative, protocol: first, at -80 mV the medium was changed from ND96 → HK → HK+acetylcholine following washout in the reversed order (HK → ND96) in order to verify successful functional expression of GIRKs. Second, the oocytes were superfused with HK0 → HK0+acetylcholine. In the case of a total loss of K^+^ selectivity and significant permeation of Na^+^ through open GIRK channels, a detectable increase in holding current by acetylcholine should have been observable in HK0. This, however, was not the case for any of the constructs tested (data not shown).

### Genetic engineering and biochemistry

The plasmids containing constructs encoding human GIRK1 N-terminally labeled with eGFP (GIRK1^eGP^), human GIRK4 and m_2_R have been previously described (20, 27). Plasmids encoding G-protein G_β2_ and G_γ1_ subunits were as described by (28). Generation of point mutations and mRNA synthesis was performed as described (27). The sequence of all generated constructs was verified by Sanger sequencing (Microsite AG; Vienna; Austria). Western blot (WB) analysis of frog oocytes was performed as described (29).

### Data normalization and statistics

In order to eliminate scatter introduced by batch-to-batch variation of protein expression between oocyte preparations, all ΔF (i.e. fluorescence intensity of the membrane - fluorescence intensity of the background) or I _total_ (I _total_ = I_HK_ + I_ACh_) measured values were normalized to ΔF or I _total_ readings of the *wt* GIRK1^eGFP^/GIRK4 heterooligomeric channel of a given experimental day and batch of oocytes. Statistical analysis of the data listed in **Table 1** and **Supplementary Tables 2-4** (and shown graphically in **Figures 2B**, **3B**, **6D**) was performed with MATLAB^®^ R2022a and with the public domain software R-4.2.1. After assessing approximate normal distribution of data (z-value of skewness and kurtosis, Q-Q plot and p value of Shapiro-Wilk test) the bootstrap-t method (2000 replicates) was utilized to obtain a robust inferential statistical characterization. In detail the R functions yuenbt(), t1waybt() and linconbt() were used to perform comparison of two independent means, one way ANOVA and multiple comparisons to a control group (Dunnett T3 procedure), respectively (30). All other experimental parameters were tested for statistically significant differences by one way ANOVA. In the case that the data were not normally distributed, Kruskal Wallis One Way Analysis of Variance was used (SPSS package in Sigmaplot 14.5; Systat Software).

**Table 1:** Statistics of P_Na+_/P_K+_ ratios.

	*P_Na+/K+_mean*	*SEM*	*N*	*p-value(WT vs. mutant)*
WT	0,021	0,000	8	
S132Y	0,020	0,001	11	> 0,999
L135I	0,022	0,001	7	> 0,999
F136L	0,022	0,001	5	> 0,999
E140M	0,021	0,001	7	> 0,999
A142T	0,021	0,001	7	> 0,999
G145A	0,075	0,003	8	*< 0,001*
R149Q	0,021	0,004	4	> 0,999
G178D	0,020	0,001	9	> 0,999
M184I	0,020	0,001	7	> 0,999
S185Y	0,021	0,001	13	> 0,999
Q186R	0,020	0,002	6	> 0,999
G4 (homo)	0,019	0,001	13	> 0,999

The value in red font color denote statistical significance.

## Results

**Figure 1:**
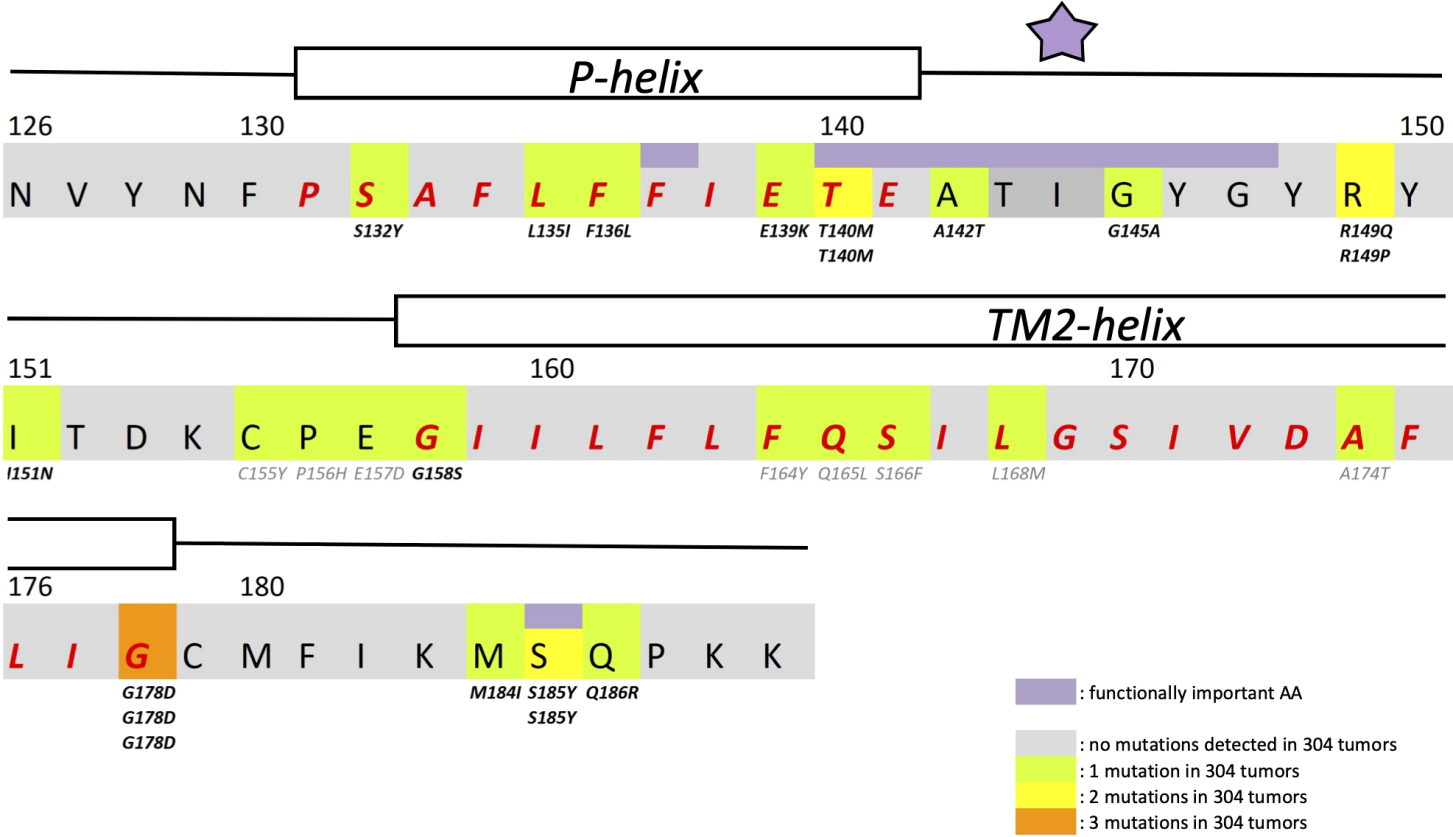
Structural details of GIRK1 subunit. Partial primary structure of the human GIRK1 subunit. Amino acid numbering according to entire full length variant 1a. Regions comprising the pore helix and the transmembrane helix 2 are indicated and the corresponding amino acids emphasized in red. Amino acids of outstanding interest are highlighted in purple (F137, ion selectivity signature (indicated by a purple asterisk) and S185). Somatic mutations detected are highlighted in *green* (1 mutation found), *yellow* (2 mutations found) and *orange* (3 mutations found). Multiple appearance of a mutation underneath an amino acid residue indicates multiple occurrence in different tumors of the sample cohort.

**Figure 2:**
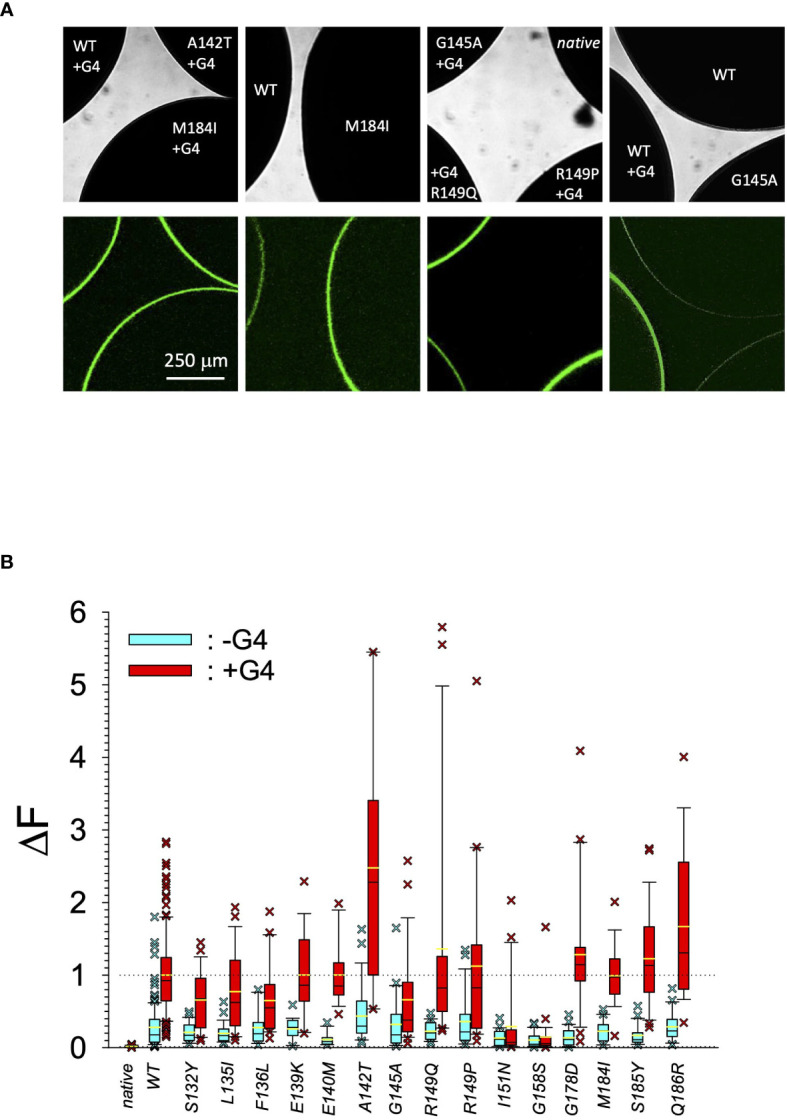
Quantitative expression of GIRK1 subunit mutations. **(A)** Original micrographs of oocytes expressing different GIRK1 constructs with and without GIRK4 coexpressed. *Upper row*: Transmission. *Lower row*: Fluorescence. **(B)** Expression levels of different GIRK1^eGFP^ subunit mutations, relative to WT. *Blue boxes*: GIRK1 mRNA injected alone. *Red boxes*: GIRK1 was coinjected with GIRK4 mRNA. Box plots comprise 25% and 75% percentiles. Median (solid black line within box) and average value (yellow line) indicated within box. Whiskers denote 10% and 90% percentiles. Oocytes having values above 90% and below 10% percentiles are shown as cross. 14 – 142 oocytes per experimental group (see **Supplementary Table 2** for exact values, numbers and statistics).

**Figure 3:**
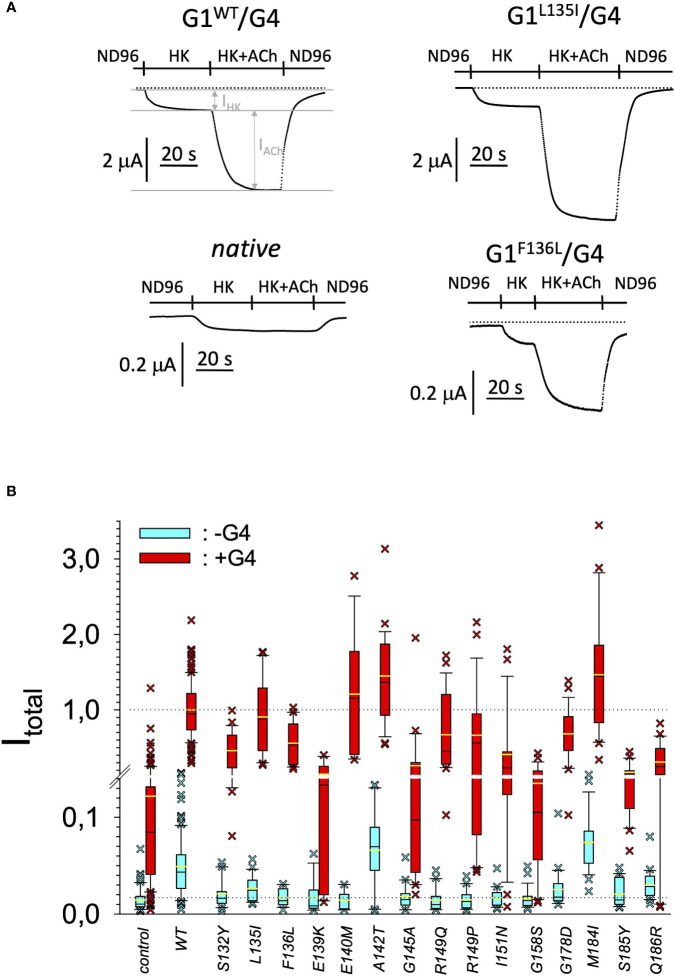
Current amplitudes produced by the different GIRK1 mutations. **(A)** Representative original TEVC current recordings, produced by different GIRK1 subunits, at a holding potential of -80 mV. Superfusion medium was exchanged as indicated above the traces. **(B)** Total current amplitudes, produced by different GIRK1^eGFP^ subunit mutations, relative to WT. *Blue boxes*: GIRK1 mRNA injected alone. *Red boxes*: GIRK1 was coinjected with GIRK4 mRNA. Box plots comprise 25% and 75% percentiles. Median (solid black line within box) and average value (yellow line) indicated within box. Whiskers denote 10% and 90% percentiles. Oocytes having values above 90% and below 10% percentiles are shown as cross. 14 – 145 oocytes per experimental group (see **Supplementary Table 3** for exact values, numbers and statistics).

**Figure 4:**
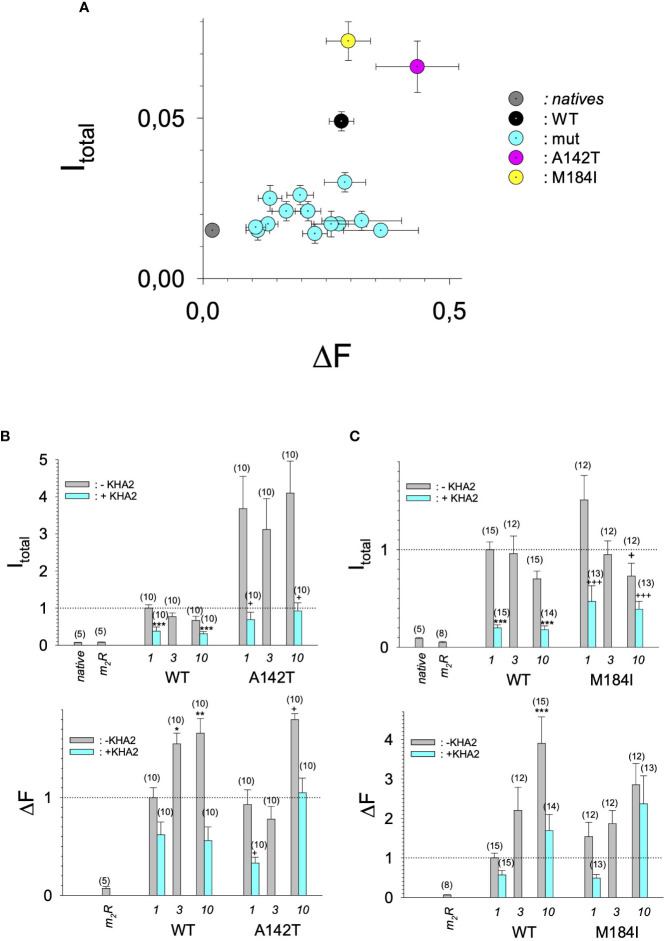
Current amplitudes versus expression levels produced by different GIRK1 mutations injected alone. **(A)** Mean values of DF versus I _total_ mean values of the different experimental groups per experimental day, without coexpression of GIRK4 subunit. Both I _total_ and DF are normalized to the levels produced by GIRK1^WT^/GIRK4 on the same experimental day and batch of oocytes. *Grey*: native oocytes, *black*: GIRK1^WT^, *purple*: GIRK1^A142T^, *yellow*: GIRK1^M184I^ and *cyan*: all the other GIRK1 subunit mutations tested. Whiskers represent SEMs. **(B)** I _total_ (*upper*) and DF values (*lower*), resulting from injection of different amounts of mRNA encoding for GIRK1^WT^ or GIRK1^A142T^, respectively (in ng at bottom of graphs). Bars represent mean values of the given experimental group. *Grey*: without coinjection of KHA2 antisense oligonucleotide, *cyan:* KHA2 antisense oligonucleotide was coinjected with mRNA. Number of oocytes given in parenthesis above the bars. Whiskers denote SEM. *, **, ***: the mean value differs statistically significant from the GIRK1^WT^ (without KHA2 antisense oligonucleotide coinjected) at the p<0.05, 0.01, 0.001 level. +,++, +++: the mean value differs statistically significant from the GIRK1^A142T^ (without KHA2 antisense oligonucleotide coinjected) at the p < 0.05, 0.01, 0.001 level. **(C)** Similar to 4B, but M184I was tested for functional GIRK1 homotetramer formation.

**Figure 5:**
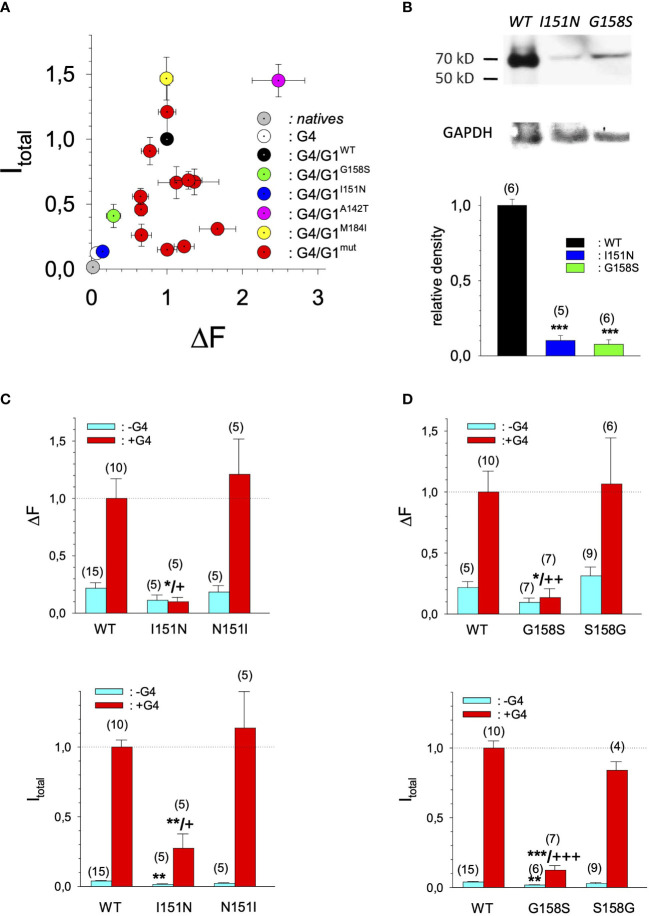
Current amplitudes versus expression levels produced by different GIRK1 mutations expressed as GIRK1/GIRK4 heterotetrameric ion channels. **(A)** Mean values of DF mean values per experimental day versus I _total_ versus mean values of the different experimental groups, produced by GIRK1/GIRK4 heterottramers. Both I _total_ and DF are normalized to the levels produced by GIRK1^WT^/GIRK4 on the same experimental day and batch of oocytes. Grey: native oocytes, *white:* GIRK4 homotetrameric ion channels, black: GIRK1^WT^/GIRK4, purple: GIRK1^A142T^/GIRK4, yellow: GIRK1^M184I^/GIRK4, *green*: GIRK1^G158S^/GIRK4, *blue*: GIRK1^I151N^/GIRK4 and *red*: all the other GIRK1 subunit mutations/GIRK4 tested. Whiskers represent SEM. **(B)** Western blots of oocyte lysates. *Upper panel*: representative lanes for oocytes expressing GIRK1^WT^/GIRK4, GIRK1^I151N^/GIRK4 and GIRK1^G158S^/GIRK4. GAPDH reference is shown underneath. *Lower panel*: Densitometric scans of GIRK1 immunoreactivity normalized to WT. *Black*: GIRK1^WT^, blue: GIRK1^I151N^ and *green*: GIRK1^G158S^. Bars represent mean values of the given experimental group. Number of lanes given in parenthesis above the bars. Whiskers denote SEM. ***: the mean value differs statistically significant from GIRK1^WT^ at the p>0.001 level. **(C)** I _total_ (*lower panel*) and DF values (*upper panel*), resulting from GIRK1^WT^, GIRK1^I151N^ and GIRK1^N151I^. *Cyan*: GIRK1 subunit mRNA conjected alone and red: GIRK1 and GIRK4 mRNAs injected. Bars represent mean values of the given experimental group. Number of oocytes given in parenthesis above the bars. Whiskers denote SEM. *,**,***: the mean value differs statistically significant from GIRK1^WT^ at the p>0.05, 0.01 and 0.001 level. +, ++, +++: the mean value differs statistically significant from GIRK1^N151I^ the at the p>0.05, 0.001, 0.001 level. **(D)** Similar to 5C but GIRK1^G158S^ was tested against GIRK1^WT^ and GIRK1^S158G^, respectively.

**Figure 6:**
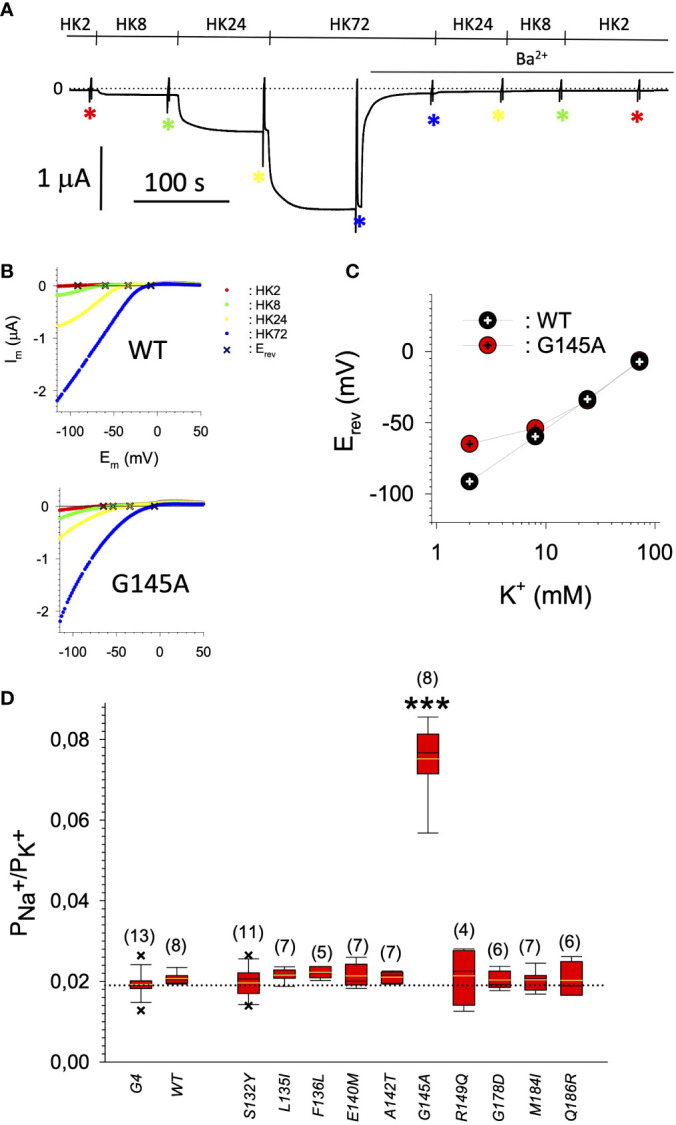
**(A)** Representative original current recording of the paradigm used for the determination of the P_Na_+/P_K+_ ratio. Holding potential was -80 mV. Superfusion with the different extracellular media is indicated above the current trace. Asterisks indicate the points in time where the voltage ramps were used for the recording of I/V relation. **(B)** Representative I/V relations for GIRK1^WT^/GIRK4 (*upper panel*) and GIRK1^G145A^/GIRK4 (*lower panel*), derived from subtraction of current recorded resulting from the voltage ramp in the presence of 2 mmole/L Ba^2+^ from the one in the absence of Ba^2+^ (*red*: HK2, *green:* HK8, *yellow*: HK24 and *blue*: HK72). Reversal potentials (E_rev_) of GIRK currents are marked by a cross.1. **(C)** Representative reversal potentials (E_rev_) measured at the different extracellular K^+^ concentrations for GIRK1^WT^/GIRK4 (*black*) and GIRK1^G145A^/GIRK4 (red). **(D)** P_Na+_/P_K+_ ratio for the different mutations tested (see **Table 1** for exact values and statistics). Bars represent mean values of the given experimental group. Number of oocytes given in parenthesis above the bars. Whiskers denote SEM. ***: the mean value differs statistically significant from GIRK1^WT^/GIRK4 at the p > 0.001 level.

In search for somatic mutations of KCNJ3 that have already been validated in malignant tumors, we trawled through the GDC Data Portal (https://portal.gdc.cancer.gov). Out of 10.202 cases of primary malignant tumors of any origin, 304 cases with somatic mutations of the KCNJ3 gene had been characterized (see **Supplementary Table 1** for a comprehensive list of the mutations found). Amongst the wide range of tumors of different origin listed in the GDC Data Portal, tumors from the uterus, skin and lung revealed the highest frequency of somatic mutations in KCNJ3, while breast cancer was least affected (see **Supplementary Figure 1**). Mutations that had occurred within the 3’ or 5’ untranslated regions, as well as synonymous mutations in the DNA that did not result in a change of the amino acid (aa) sequence, were not further considered. The same was done with mutations that led to a frameshift, resulting in truncated and hence, most likely, non-functional protein. The position of the remaining missense mutations in the primary structure of GIRK1 that comprises 501 aa’s is shown in **Supplementary Figure 2**. We focused our efforts on a region between the first and the second transmembrane helix (TM), that is of vital importance for ion selectivity and comprises the pore helix and the ion selectivity signature (31). Somatic mutations of amino acids within or near the corresponding region of the GIRK4 subunit have already been shown to render GIRK ion channels unselective for K^+^ over Na^+^ and to be important for genesis of benign adenomas of the adrenal cortex (23). Additional incitement to study this region came from the fact that several somatic mutations clustered around F137. Mutation F137S has been produced so far exclusively in the laboratory and is known to result in GIRK1 subunits that are able to form functional homooligomeric ion channels (32). Mutations in a small region at the cytosolic end of TM2 were also studied, since S185 has been identified to be important for Proteinkinase-C (PKC) regulation of GIRK1 (33) and we found somatic mutations directly at and in the neighborhood of S185 (see **Figure 1** for an overview on the region and the amino acids that have been studied). Protein expression of the different mutants in Xenopus oocytes was studied *via* the eGFP tagged GIRK1 subunit and quantitative confocal laser scan microscopy (cLSM). Original micrographs can be seen in **Figure 2A**. The average amount of protein expressed, with and without the GIRK4 subunit coexpressed, is shown in **Figure 2B** (see **Supplementary Table 2** for numerical values and statistics). Clearly, the amount of fluorescence is above background (i.e. native, uninjected oocytes) and these values are, with few exceptions, statistically significant already for the GIRK1 subunit expressed alone. The fluorescence values of GIRK1 subunits coinjected with GIRK4 are generally larger, indicating that expression of protein near and/or at the plasma membrane is more pronounced when GIRK1 becomes coexpressed along with GIRK4. Even I151N and G158S, the mutants exerting the lowest expression levels among all mutants, show low, but detectable and statistically significant fluorescence. Function of the different mutants as GIRK ion channels, with and without GIRK4 coexpressed, was assessed in parallel and on the same oocytes by the Two Electrode Voltage Clamp technique (see **Figure 3A** for examples of original recordings). As a measure of functionality of a given mutation, we chose total current (i.e.: basal current plus current induced by coexpressed muscarinic type 2 receptors (m_2_Rs)), rather than the current induced by acetylcholine. The rationale is that affinity/activation of the GIRK ion channel formed for/by the G-protein βγ subunit may have been possibly altered by a single mutation and therefore the basal current (I_HK_), induced by endogenous G_β/γ_subunits, may be larger (or smaller) at the cost of the current induced by acetylcholine (I_ACh_), while the total current (I _total_ = I_HK_ + I_ACh_) remains comparable to WT. In heterotetrameric ion channels comprising GIRK1/GIRK4 subunits, ion currents were sufficiently big to allow a reliable determination of the I_ACh_/I _total_ ratio. The majority of mutations featured I_ACh_/I _total_ ratios, similar to GIRK1^WT^/GIRK4. In the case of I151N and S185Y I_ACh_/I _total_ ratios were statistically significantly increased (I151N) or reduced (S185Y), respectively. Since I151N and S185Y result in small currents even when coexpressed with GIRK4, further studies are needed to determine whether these mutations compromise the channels’ activation by the G-protein βγ subunits (see **Supplementary Figure 4**; **Supplementary Table 4** for exact values and statistics). Inspection of the total current magnitudes produced by GIRK1 subunits alone shows that most mutants are not able to produce current, when injected alone (**Figure 3B**, **Supplementary Table 3**). A closer look at the correlation between expression and function of GIRK1 subunits, injected alone, reveals that most mutations do express to some extent, in magnitude similar or smaller than WT, but total current is reduced or absent (**Figure 4A**). Two mutations, namely A142T and M184I, stand out, as both fluorescence and total current surpass WT (**Figure 4A**). WT GIRK1 subunits are known to be uncapable to form functional homooligomeric ion channels (24). Therefore, the marginal total current observed with WT alone (approx. 5% of the current produced by WT plus GIRK4) is due to incomplete knockdown of the endogenous GIRK5 subunit in the frog oocyte by the antisense oligonucleotide. For A142T and M184I, however, the excessive current may indicate the gain of ability to form functional homotetrameric ion channels. Therefore, we tested A142T and M184I for the ability to form homooligomeric GIRK channels. Increasing amounts of GIRK1 mRNA, with and without the antisense oligonucleotide, were injected and both total current amplitudes and fluorescence were compared to WT (**Figures 4B, C**). In the case of WT, as expected, expression increased to some degree with the amount of mRNA injected. Expression and/or localization to or close to the plasma membrane is facilitated by the endogenous GIRK5 subunit as evidenced by reduced fluorescence levels upon oligonucleotide coinjection. Total current amplitudes were greatly and statistically significantly reduced upon coinjection of the antisense oligonucleotide, but did not increase with the amount of mRNA injected. This matches exactly the expectation when endogenous GIRK5 subunit is rate limiting for functional ion channel formation and the formation of functional GIRK1 homooligomeric ion channels is not possible. The small, but detectable, ion current is therefore entirely produced by heterooligomeric ion channels, containing endogenous GIRK5 that was left over from antisense oligonucleotide knockdown. A similar behavior was observed for both A142T and M184I, revealing that these mutants are unable to form homomers and require GIRK4 for the formation of functional ion channels, alike WT. In the case of A142T, current levels, but not fluorescence, were generally increased at all the mRNA concentrations tested when compared to WT indicating that A142T may represent a mutation with increased open probability relative to WT. We conclude that none of the mutations tested is able to form functional homomeric ion channels and the rather tiny, if any, amounts of currents observed were due to incomplete knockdown of the endogenous GIRK5 subunit by the antisense oligonucleotide. A survey of total current levels obtained by the constructs, when GIRK4 subunits are coexpressed, reveals that most are able to form functional heteromeric GIRK1/GIRK4 ion channels. Already at the amount of mRNA injected for this experimental paradigm, S132Y, L135I, F136L, E140M, A142T, R149Q, R149P, G178D and M184I give currents in the range of WT+GIRK4 and readily above background (**Figure 3B**; see **Supplementary Table 3** for exact values and statistics). Upon injection of higher amounts of mRNA, E139K, G145A, I151N, G158S, S185Y and Q186R give clearly detectable currents when coexpressed with GIRK4 and hence are also functional (see **Supplementary Figure 4**). A thorough survey of the correlation of the amount of fluorescence with the total current measured reveals that, upon GIRK4 coexpression, most mutants give expression levels similar to WT+GIRK4 and similar or reduced amounts of total current (**Figure 5A**). Notably are I151N and G158S, that give detectable currents despite marginal expression levels, suggesting an abnormally high rate of function per protein expressed. Western blot analysis confirmed that detectable but greatly reduced amounts of protein, compared to WT+GIRK4, are synthesized by the oocytes (**Figure 5B**). In order to validate that the constructs containing I151N or G158S are not compromised by any mutation that had eventually occurred during site directed mutagenesis, but had not been detected by us, the respective inserts were mutated back to yield WT primary structure. Fluorescence and total current of WT, the mutants (I151N or G158S) and the backmutated constructs (N151I or S158G) were compared (with and without GIRK4 coexpressed) and it turned out that WT and N151I (or S158G) are practically indistinguishable (**Figures 5C, D**). Since I151N and G158S stick out by measurable current at miniscule expression, they were also checked for proficiency to form homooligomeric ion channels. This was, however, not the case (**Supplementary Figure 3**). Eventually we arrive at the conclusion that I151N and G158S represent mutations with extremely low expression levels of GIRK1 protein but, relative to the amount of protein, overproportionate activity as ion channels upon coexpression with GIRK4. Since the mutations studied are located within a region that is considered to be important for ion selectivity, reversal potentials of currents through GIRK1/GIRK4 heterotetramers were measured at different extracellular K^+^ concentrations and the ratio of permeability coefficients for K^+^ and Na^+^ (P_Na+_/P_K+_) was calculated (**Figure 6A, B**). Mutations that gave detectable currents, that were too small, even at the highest amounts of mRNA possible to inject, to allow precise determination of P_Na+_/P_K+_ (E139K, R149P, G158S, G178D and S185Y), were subjected to a simplified protocol: First, basal and acetylcholine induced currents were measured (as exemplified in **Figure 3A**) under regular conditions, followed by a recapitulation where the K^+^ in the HK solution was entirely replaced by Na^+^. Since no detectable currents were elicited by acetylcholine during the recapitulation with any of the constructs listed above, we conclude that these mutations do not suffer from impairment of ion selectivity (not shown). Exact determination of P_Na+_/P_K+_ was possible for WT and mutations S132Y, L135I, F136L, E140M, A142T, G145A, R149Q, I151N, M184I and Q186R. The results are shown in **Figure 6C**. It becomes apparent that G145A has markedly increased P_Na+_/P_K+_, compared to WT heterooligomeric. P_Na+_/P_K+_ ratio of GIRK4 homooligomeric ion channels and of the other mutations studied remained unchanged (see **Table 1** for exact values and statistics).

## Discussion

Our study clearly shows that somatic missense mutations of GIRK1, validated in malignant tumors, can have profound effects on both expression and on the function of G-protein activated inwardly rectifying potassium channels. Facing these alterations of phenotype, it is of interest to contemplate our knowledge of GIRK ion channel disfunction in humans. Inherited and/or somatic mutations of the genes encoding GIRKs have been identified to bring along several implications for neuronal, endocrine and cardiac conditions (4, 23, 34). In the case of primary aldosteronism (PA), the most frequently occurring manifestation of secondary hypertension, a substantial fraction of cases is brought about by benign aldosterone producing adenomas caused by mutations within a region of KCNJ5 that is constraint to the pore and selectivity filter of the GIRK4 subunit. The results are GIRK4 subunits containing functional ion channels that are not able to discriminate between Na^+^ and K^+^ ions, featuring reversed impact on cellular physiology (35). Out of the 16 mutations studied, we found G145A, that is located directly within the ion selectivity filter of GIRK1 (**Figure 1**), to result in restrained P_Na+_/P_K+_ of GIRK1/GIRK4 channels. This effect on ion discrimination is detectable, but small and far from a complete loss of ion discrimination. Hence it is questionable, whether the observed *approx. 3x* increased, but still humble, Na^+^ permeability is able to play a relevant role in pathophysiology. In this context it is of particular interest that a recent study has shown that somatic mutations of KCNJ5 that lead to abnormal expression and/or activation of GIRKs, are also able to induce PA (26), alike their well-studied counterparts that result in a complete loss of ion selectivity. The majority of mutations studied here exerted phenotypes that can be roughly classified into the following categories: (i) normal/reduced expression accompanied by reduced/absent function (S132Y, F136L, E139K, G145A, R149Q, R149P, G178D, S185Y, Q186R), (ii) normal/increased expression as well as increased function (E140M, A142T, M184I) and (iii) miniscule expression but increased function, relative to expression levels (I151N, G158S). Abnormally high expression levels, but not somatic mutation, of GIRK1 has been shown to play a role in the generation and progression of breast cancer (16–22). As shown here, primary tumors of the breast turned out to be the least affected by somatic mutations of KCNJ3, when compared to other malignancies. Apparently, overexpression of GIRK1 results in similar functional consequences as gain of function *via* somatic mutation(s) will bring about. Therefore, gain of function mutations, identical or similar to the ones identified here, might well be involved in genesis and progression of malignancies other than breast, in tissues that exert a high rate of occurrence of somatic mutations of KCNJ3. We emphasize that groups (ii) and (iii) are therefore likely to have profound effects on pathophysiological conditions. Somatic mutations that have normal or reduced levels of expression and reduced or absent function are less likely to promote genesis and/or progression of cancer and are hence categorized into a single group, i.e. (i). It should be emphasized that the functional spectrum of the validated somatic mutations is broad. This spectrum comprises mutations in the untranslated regions, that potentially might have effects at the transcriptional level, silent mutations, that obviously have at most minor effects, loss of function mutations (mostly mutations resulting in a frameshift, but also category (i)) and gain of function mutations, as identified here [categories (ii) and (iii)].

In order to understand, whether somatic mutations in the KCNJ3 gene are indeed important in clinical settings, will require more, careful and targeted screening and compilation of somatic mutations in tumor samples in the context of histological parameters as well as cancer progression accompanied by pursuing studies in human cancer as well as cancer progenitor cells, in the future.

## Data availability statement

The raw data supporting the conclusions of this article will be made available by the authors, without undue reservation.

## Ethics statement

The animal study was reviewed and approved by Ethics committee of the Medical University of Graz.

## Author contributions

BP, SS, SL, TR, AH, AG, and WS were involved in planning the project, discussion of results and made experiments. AS was involved in planning the project, discussion of results and performing researches in the GDC database. DP, KZ-P, SJ, and TB were involved in planning the project and discussion of results. KZ-P and DP performed statistical analyses with Matlab. WS prepared the figures and wrote the manuscript. All authors contributed to the article and approved the submitted version.

## Conflict of interest

The authors declare that the research was conducted in the absence of any commercial or financial relationships that could be construed as a potential conflict of interest.

## Publisher’s note

All claims expressed in this article are solely those of the authors and do not necessarily represent those of their affiliated organizations, or those of the publisher, the editors and the reviewers. Any product that may be evaluated in this article, or claim that may be made by its manufacturer, is not guaranteed or endorsed by the publisher.
