# Supplementary material for: Consequences of somatic mutations of GIRK1 detected in primary malign tumors on expression and function of G-protein activated, inwardly rectifying, K+ channels
Source: Front Oncol. 2022 Oct 31;12:998907. doi: 10.3389/fonc.2022.998907 (PMC9724741; doi:10.3389/fonc.2022.998907)
Supplement: Supplementary file 1 [file DataSheet_1.pdf]

Supplementary Table 1.:

	DNA Change	*Type*	*Consequences*	*# Affected Cases in Cohort*
	chr2:g.154855625delT	Deletion	3 Prime UTR KCNJ3	6 / 304 1.97%
	chr2:g.154709779C>T	Substitution	Synonymous KCNJ3 F293F	4 / 304 1.32%
	chr2:g.154854785delT	Deletion	Frameshift KCNJ3 P329Lfs*2	4 / 304 1.32%
	chr2:g.154855732delTG	Deletion	3 Prime UTR KCNJ3	4 / 304 1.32%
	chr2:g.154699308G>A	Substitution	Missense KCNJ3 G178D	3 / 304 0.99%
	chr2:g.154855617delAA	Deletion	3 Prime UTR KCNJ3	3 / 304 0.99%
	chr2:g.154699189C>T	Substitution	Synonymous KCNJ3 I138I	3 / 304 0.99%
	chr2:g.154709629C>T	Substitution	Synonymous KCNJ3 F243F	2 / 304 0.66%
	chr2:g.154698983G>T	Substitution	Missense KCNJ3 D70Y	2 / 304 0.66%
	chr2:g.154709654G>A	Substitution	Missense KCNJ3 D252N	2 / 304 0.66%
	chr2:g.154699194C>T	Substitution	Missense KCNJ3 T140M	2 / 304 0.66%
	chr2:g.154855140G>T	Substitution	Missense KCNJ3 G445C	2 / 304 0.66%
	chr2:g.154855052A>G	Substitution	Synonymous KCNJ3 K415K	2 / 304 0.66%
	chr2:g.154709722C>T	Substitution	Synonymous KCNJ3 I274I	2 / 304

				0.66%
☐	chr2:g.154855137C>T	Substitution	Missense KCNJ3 P444S	2 / 304<br>0.66%
☐	chr2:g.154854792C>T	Substitution	Missense KCNJ3 P329S	2 / 304<br>0.66%
☐	chr2:g.154709696T>C	Substitution	Missense KCNJ3 S266P	2 / 304<br>0.66%
☐	chr2:g.154709690C>A	Substitution	Missense KCNJ3 L264I	2 / 304<br>0.66%
☐	chr2:g.154855055C>A	Substitution	Synonymous KCNJ3 L416L	2 / 304<br>0.66%
☐	chr2:g.154855302C>T	Substitution	Missense KCNJ3 R499C	2 / 304<br>0.66%
☐	chr2:g.154855101G>A	Substitution	Missense KCNJ3 D432N	2 / 304<br>0.66%
☐	chr2:g.154699329C>A	Substitution	Missense KCNJ3 S185Y	2 / 304<br>0.66%
☐	chr2:g.154855972C>A	Substitution	3 Prime UTR KCNJ3	2 / 304<br>0.66%
☐	chr2:g.154854771C>A	Substitution	Missense KCNJ3 L322I	2 / 304<br>0.66%
☐	chr2:g.154698981C>T	Substitution	Missense KCNJ3 S69L	2 / 304<br>0.66%
☐	chr2:g.154855123G>A	Substitution	Missense KCNJ3 R439Q	2 / 304<br>0.66%
☐	chr2:g.154854881G>A	Substitution	Synonymous KCNJ3 V358V	2 / 304<br>0.66%
☐	chr2:g.154855046C>T	Substitution	Synonymous KCNJ3 P413P	2 / 304<br>0.66%

☐	chr2:g.154855119C>T	Substitution	Stop Gained KCNJ3 Q438*	2 / 304<br>0.66%
☐	chr2:g.154699345C>T	Substitution	Synonymous KCNJ3 R190R	2 / 304<br>0.66%
☐	chr2:g.154854745G>A	Substitution	Missense KCNJ3 R313Q	2 / 304<br>0.66%
☐	chr2:g.154855097G>T	Substitution	Missense KCNJ3 L430F	2 / 304<br>0.66%
☐	chr2:g.154698789G>A	Substitution	Missense KCNJ3 R5Q	2 / 304<br>0.66%
☐	chr2:g.154699091G>A	Substitution	Missense KCNJ3 A106T	2 / 304<br>0.66%
☐	chr2:g.154709757G>A	Substitution	Missense KCNJ3 R286Q	2 / 304<br>0.66%
☐	chr2:g.154699071C>T	Substitution	Missense KCNJ3 A99V	2 / 304<br>0.66%
☐	chr2:g.154709621G>A	Substitution	Missense KCNJ3 G241S	2 / 304<br>0.66%
☐	chr2:g.154855712G>A	Substitution	3 Prime UTR KCNJ3	1 / 304<br>0.33%
☐	chr2:g.154854931C>A	Substitution	Missense KCNJ3 A375D	1 / 304<br>0.33%
☐	chr2:g.154855764C>T	Substitution	3 Prime UTR KCNJ3	1 / 304<br>0.33%
☐	chr2:g.154855303G>A	Substitution	Missense KCNJ3 R499H	1 / 304<br>0.33%
☐	chr2:g.154699264C>A	Substitution	Synonymous KCNJ3 L163L	1 / 304<br>0.33%
☐	chr2:g.154699309C>A	Substitution	Synonymous KCNJ3 G178G	1 / 304

				0.33%
☐	chr2:g.154855024T>A	Substitution	Missense KCNJ3 I406N	1 / 304<br>0.33%
☐	chr2:g.154699276C>T	Substitution	Synonymous KCNJ3 I167I	1 / 304<br>0.33%
☐	chr2:g.154855303G>T	Substitution	Missense KCNJ3 R499L	1 / 304<br>0.33%
☐	chr2:g.154855329G>T	Substitution	3 Prime UTR KCNJ3	1 / 304<br>0.33%
☐	chr2:g.154699431G>A	Substitution	Missense KCNJ3 R219H	1 / 304<br>0.33%
☐	chr2:g.154855254A>G	Substitution	Missense KCNJ3 R483G	1 / 304<br>0.33%
☐	chr2:g.154699404C>T	Substitution	Missense KCNJ3 T210M	1 / 304<br>0.33%
☐	chr2:g.154855813C>T	Substitution	3 Prime UTR KCNJ3	1 / 304<br>0.33%
☐	chr2:g.154698770C>T	Substitution	5 Prime UTR KCNJ3	1 / 304<br>0.33%
☐	chr2:g.154699053T>A	Substitution	Missense KCNJ3 V93E	1 / 304<br>0.33%
☐	chr2:g.154854866C>A	Substitution	Synonymous KCNJ3 T353T	1 / 304<br>0.33%
☐	chr2:g.154709660G>T	Substitution	Missense KCNJ3 G254C	1 / 304<br>0.33%
☐	chr2:g.154854843C>T	Substitution	Missense KCNJ3 H346Y	1 / 304<br>0.33%
☐	chr2:g.154855137C>A	Substitution	Missense KCNJ3 P444T	1 / 304<br>0.33%

☐	chr2:g.154855222C>A	Substitution	Missense KCNJ3 P472Q	1 / 304<br>0.33%
☐	chr2:g.154699249C>A	Substitution	Synonymous KCNJ3 G158G	1 / 304<br>0.33%
☐	chr2:g.154854900C>T	Substitution	Missense KCNJ3 L365F	1 / 304<br>0.33%
☐	chr2:g.154855269C>T	Substitution	Missense KCNJ3 L488F	1 / 304<br>0.33%
☐	chr2:g.154855161G>A	Substitution	Missense KCNJ3 V452I	1 / 304<br>0.33%
☐	chr2:g.154698889G>T	Substitution	Synonymous KCNJ3 V38V	1 / 304<br>0.33%
☐	chr2:g.154854838A>C	Substitution	Missense KCNJ3 Q344P	1 / 304<br>0.33%
☐	chr2:g.154709682A>C	Substitution	Missense KCNJ3 Q261P	1 / 304<br>0.33%
☐	chr2:g.154855097delG	Deletion	Frameshift KCNJ3 G431Efs*5	1 / 304<br>0.33%
☐	chr2:g.154854910C>T	Substitution	Missense KCNJ3 S368L	1 / 304<br>0.33%
☐	chr2:g.154854948G>T	Substitution	Stop Gained KCNJ3 E381*	1 / 304<br>0.33%
☐	chr2:g.154699391G>A	Substitution	Missense KCNJ3 D206N	1 / 304<br>0.33%
☐	chr2:g.154855019G>A	Substitution	Synonymous KCNJ3 Q404Q	1 / 304<br>0.33%
☐	chr2:g.154709745A>G	Substitution	Missense KCNJ3 D282G	1 / 304<br>0.33%
☐	chr2:g.154698941C>A	Substitution	Missense KCNJ3 Q56K	1 / 304

			0.33%
chr2:g.154855156A>T	Substitution	Missense KCNJ3 K450I	1 / 304 0.33%
chr2:g.154698871C>A	Substitution	Missense KCNJ3 D32E	1 / 304 0.33%
chr2:g.154698793G>A	Substitution	Synonymous KCNJ3 R6R	1 / 304 0.33%
chr2:g.154855000A>G	Substitution	Missense KCNJ3 K398R	1 / 304 0.33%
chr2:g.154855186C>A	Substitution	Missense KCNJ3 S460Y	1 / 304 0.33%
chr2:g.154855090A>G	Substitution	Missense KCNJ3 Y428C	1 / 304 0.33%
chr2:g.154698805C>A	Substitution	Missense KCNJ3 D10E	1 / 304 0.33%
chr2:g.154709624G>A	Substitution	Missense KCNJ3 E242K	1 / 304 0.33%
chr2:g.154855638T>G	Substitution	3 Prime UTR KCNJ3	1 / 304 0.33%
chr2:g.154709706C>T	Substitution	Missense KCNJ3 T269I	1 / 304 0.33%
chr2:g.154699239G>A	Substitution	Missense KCNJ3 C155Y	1 / 304 0.33%
chr2:g.154855124A>G	Substitution	Synonymous KCNJ3 R439R	1 / 304 0.33%
chr2:g.154854903C>A	Substitution	Missense KCNJ3 L366I	1 / 304 0.33%
chr2:g.154854981C>T	Substitution	Synonymous KCNJ3 L392L	1 / 304 0.33%

☐	chr2:g.154699242C>A	Substitution	Missense KCNJ3 P156H	1 / 304<br>0.33%
☐	chr2:g.154699201C>A	Substitution	Synonymous KCNJ3 A142A	1 / 304<br>0.33%
☐	chr2:g.154698875C>G	Substitution	Missense KCNJ3 Q34E	1 / 304<br>0.33%
☐	chr2:g.154698746G>A	Substitution	5 Prime UTR KCNJ3	1 / 304<br>0.33%
☐	chr2:g.154855463G>A	Substitution	3 Prime UTR KCNJ3	1 / 304<br>0.33%
☐	chr2:g.154856419C>G	Substitution	3 Prime UTR KCNJ3	1 / 304<br>0.33%
☐	chr2:g.154854911G>A	Substitution	Synonymous KCNJ3 S368S	1 / 304<br>0.33%
☐	chr2:g.154698763G>A	Substitution	5 Prime UTR KCNJ3	1 / 304<br>0.33%
☐	chr2:g.154855088C>A	Substitution	Synonymous KCNJ3 A427A	1 / 304<br>0.33%
☐	chr2:g.154699294C>T	Substitution	Synonymous KCNJ3 D173D	1 / 304<br>0.33%
☐	chr2:g.154698895G>A	Substitution	Synonymous KCNJ3 K40K	1 / 304<br>0.33%
☐	chr2:g.154699269A>T	Substitution	Missense KCNJ3 Q165L	1 / 304<br>0.33%
☐	chr2:g.154699106G>A	Substitution	Missense KCNJ3 D111N	1 / 304<br>0.33%
☐	chr2:g.154855297C>A	Substitution	Missense KCNJ3 S497Y	1 / 304<br>0.33%
☐	chr2:g.154854828G>T	Substitution	Missense KCNJ3 D341Y	1 / 304

				0.33%
☐	chr2:g.154855167delA	Deletion	Frameshift KCNJ3 T455Pfs*9	1 / 304<br>0.33%
☐	chr2:g.154699178C>A	Substitution	Missense KCNJ3 L135I	1 / 304<br>0.33%
☐	chr2:g.154855175C>T	Substitution	Synonymous KCNJ3 T456T	1 / 304<br>0.33%
☐	chr2:g.154855820A>C	Substitution	3 Prime UTR KCNJ3	1 / 304<br>0.33%
☐	chr2:g.154709635C>A	Substitution	Synonymous KCNJ3 P245P	1 / 304<br>0.33%
☐	chr2:g.154699145G>A	Substitution	Missense KCNJ3 V124M	1 / 304<br>0.33%
☐	chr2:g.154855058G>C	Substitution	Missense KCNJ3 L417F	1 / 304<br>0.33%
☐	chr2:g.154855092A>T	Substitution	Missense KCNJ3 S429C	1 / 304<br>0.33%
☐	chr2:g.154709672G>T	Substitution	Missense KCNJ3 G258W	1 / 304<br>0.33%
☐	chr2:g.154698931G>T	Substitution	Synonymous KCNJ3 R52R	1 / 304<br>0.33%
☐	chr2:g.154699272C>T	Substitution	Missense KCNJ3 S166F	1 / 304<br>0.33%
☐	chr2:g.154699066C>T	Substitution	Synonymous KCNJ3 F97F	1 / 304<br>0.33%
☐	chr2:g.154854734T>G	Substitution	Synonymous KCNJ3 T309T	1 / 304<br>0.33%
☐	chr2:g.154855852T>C	Substitution	3 Prime UTR KCNJ3	1 / 304<br>0.33%

chr2:g.154699227T>A	Substitution	Missense KCNJ3 I151N	1 / 304<br>0.33%
chr2:g.154854818C>A	Substitution	Missense KCNJ3 F337L	1 / 304<br>0.33%
chr2:g.154699228C>A	Substitution	Synonymous KCNJ3 I151I	1 / 304<br>0.33%
chr2:g.154855046_154855047insA	Insertion	Frameshift KCNJ3 L416Tfs*39	1 / 304<br>0.33%
chr2:g.154699332A>G	Substitution	Missense KCNJ3 Q186R	1 / 304<br>0.33%
chr2:g.154854729A>G	Substitution	Missense KCNJ3 M308V	1 / 304<br>0.33%
chr2:g.154855556A>C	Substitution	3 Prime UTR KCNJ3	1 / 304<br>0.33%
chr2:g.154855112G>A	Substitution	Missense KCNJ3 M435I	1 / 304<br>0.33%
chr2:g.154855122C>T	Substitution	Stop Gained KCNJ3 R439*	1 / 304<br>0.33%
chr2:g.154698912T>G	Substitution	Missense KCNJ3 F46C	1 / 304<br>0.33%
chr2:g.154698826A>G	Substitution	Synonymous KCNJ3 T17T	1 / 304<br>0.33%
chr2:g.154709666A>T	Substitution	Missense KCNJ3 S256C	1 / 304<br>0.33%
chr2:g.154698867A>T	Substitution	Missense KCNJ3 Q31L	1 / 304<br>0.33%
chr2:g.154855263G>A	Substitution	Missense KCNJ3 G486R	1 / 304<br>0.33%
chr2:g.154709716C>T	Substitution	Synonymous KCNJ3 H272H	1 / 304

				0.33%
☐	chr2:g.154699450C>T	Substitution	Synonymous KCNJ3 S225S	1 / 304 0.33%
☐	chr2:g.154698912T>C	Substitution	Missense KCNJ3 F46S	1 / 304 0.33%
☐	chr2:g.154699277C>A	Substitution	Missense KCNJ3 L168M	1 / 304 0.33%
☐	chr2:g.154854753T>A	Substitution	Missense KCNJ3 Y316N	1 / 304 0.33%
☐	chr2:g.154855103_154855104insT	Insertion	Frameshift KCNJ3 L433Ffs*22	1 / 304 0.33%
☐	chr2:g.154855173A>C	Substitution	Missense KCNJ3 T456P	1 / 304 0.33%
☐	chr2:g.154854922T>A	Substitution	Missense KCNJ3 I372K	1 / 304 0.33%
☐	chr2:g.154698971C>T	Substitution	Missense KCNJ3 R66C	1 / 304 0.33%
☐	chr2:g.154855045C>T	Substitution	Missense KCNJ3 P413L	1 / 304 0.33%
☐	chr2:g.154855384C>A	Substitution	3 Prime UTR KCNJ3	1 / 304 0.33%
☐	chr2:g.154699267C>T	Substitution	Synonymous KCNJ3 F164F	1 / 304 0.33%
☐	chr2:g.154709796T>C	Substitution	Missense KCNJ3 L299P	1 / 304 0.33%
☐	chr2:g.154855617delA	Deletion	3 Prime UTR KCNJ3	1 / 304 0.33%
☐	chr2:g.154855324C>A	Substitution	3 Prime UTR KCNJ3	1 / 304 0.33%

☐	chr2:g.154856074T>G	Substitution	3 Prime UTR KCNJ3	1 / 304<br>0.33%
☐	chr2:g.154709607G>T	Substitution	Missense KCNJ3 R236L	1 / 304<br>0.33%
☐	chr2:g.154699264C>G	Substitution	Synonymous KCNJ3 L163L	1 / 304<br>0.33%
☐	chr2:g.154854842C>G	Substitution	Missense KCNJ3 F345L	1 / 304<br>0.33%
☐	chr2:g.154855186C>T	Substitution	Missense KCNJ3 S460F	1 / 304<br>0.33%
☐	chr2:g.154854771C>T	Substitution	Missense KCNJ3 L322F	1 / 304<br>0.33%
☐	chr2:g.154854784_154854785insT	Insertion	Frameshift KCNJ3 P329Sfs*11	1 / 304<br>0.33%
☐	chr2:g.154855152G>T	Substitution	Stop Gained KCNJ3 E449*	1 / 304<br>0.33%
☐	chr2:g.154856365C>A	Substitution	3 Prime UTR KCNJ3	1 / 304<br>0.33%
☐	chr2:g.154855891A>G	Substitution	3 Prime UTR KCNJ3	1 / 304<br>0.33%
☐	chr2:g.154699221G>A	Substitution	Missense KCNJ3 R149Q	1 / 304<br>0.33%
☐	chr2:g.154854910C>A	Substitution	Stop Gained KCNJ3 S368*	1 / 304<br>0.33%
☐	chr2:g.154709606C>T	Substitution	Missense KCNJ3 R236W	1 / 304<br>0.33%
☐	chr2:g.154698815G>T	Substitution	Missense KCNJ3 V14L	1 / 304<br>0.33%
☐	chr2:g.154699105C>T	Substitution	Synonymous KCNJ3 G110G	1 / 304

0.33%

chr2:g.154699010A>C	Substitution	Missense KCNJ3 K79Q	1 / 304 0.33%
chr2:g.154855085A>G	Substitution	Synonymous KCNJ3 K426K	1 / 304 0.33%
chr2:g.154855367T>C	Substitution	3 Prime UTR KCNJ3	1 / 304 0.33%
chr2:g.154854731G>C	Substitution	Missense KCNJ3 M308I	1 / 304 0.33%
chr2:g.154699051C>G	Substitution	Synonymous KCNJ3 T92T	1 / 304 0.33%
chr2:g.154709704C>T	Substitution	Synonymous KCNJ3 L268L	1 / 304 0.33%
chr2:g.154699449C>T	Substitution	Missense KCNJ3 S225F	1 / 304 0.33%
chr2:g.154854812G>T	Substitution	Missense KCNJ3 E335D	1 / 304 0.33%
chr2:g.154698901G>C	Substitution	Missense KCNJ3 K42N	1 / 304 0.33%
chr2:g.154699374C>T	Substitution	Missense KCNJ3 A200V	1 / 304 0.33%
chr2:g.154854934T>C	Substitution	Missense KCNJ3 I376T	1 / 304 0.33%
chr2:g.154709812A>T	Substitution	Missense KCNJ3 E304D	1 / 304 0.33%
chr2:g.154699216C>G	Substitution	Synonymous KCNJ3 G147G	1 / 304 0.33%
chr2:g.154709779C>A	Substitution	Missense KCNJ3 F293L	1 / 304 0.33%

☐	chr2:g.154855008T>A	Substitution	Missense KCNJ3 S401T	1 / 304<br>0.33%
☐	chr2:g.154856050C>A	Substitution	3 Prime UTR KCNJ3	1 / 304<br>0.33%
☐	chr2:g.154699019T>C	Substitution	Missense KCNJ3 W82R	1 / 304<br>0.33%
☐	chr2:g.154854965G>A	Substitution	Synonymous KCNJ3 V386V	1 / 304<br>0.33%
☐	chr2:g.154855038delGACTTTCC...	Deletion	Frameshift KCNJ3 D411Lfs*25	1 / 304<br>0.33%
☐	chr2:g.154856027C>A	Substitution	3 Prime UTR KCNJ3	1 / 304<br>0.33%
☐	chr2:g.154698938G>C	Substitution	Missense KCNJ3 V55L	1 / 304<br>0.33%
☐	chr2:g.154699246G>T	Substitution	Missense KCNJ3 E157D	1 / 304<br>0.33%
☐	chr2:g.154698872C>G	Substitution	Missense KCNJ3 P33A	1 / 304<br>0.33%
☐	chr2:g.154854870C>A	Substitution	Missense KCNJ3 P355T	1 / 304<br>0.33%
☐	chr2:g.154856206C>T	Substitution	3 Prime UTR KCNJ3	1 / 304<br>0.33%
☐	chr2:g.154699221G>C	Substitution	Missense KCNJ3 R149P	1 / 304<br>0.33%
☐	chr2:g.154698740C>T	Substitution	5 Prime UTR KCNJ3	1 / 304<br>0.33%
☐	chr2:g.154709780G>A	Substitution	Missense KCNJ3 E294K	1 / 304<br>0.33%
☐	chr2:g.154855024T>G	Substitution	Missense KCNJ3 I406S	1 / 304

			0.33%
chr2:g.154709701_154709702insCTCACAA	Insertion	Frameshift KCNJ3 I270Tfs*15	1 / 304 0.33%
chr2:g.154699346G>A	Substitution	Missense KCNJ3 A191T	1 / 304 0.33%
chr2:g.154699090C>G	Substitution	Missense KCNJ3 I105M	1 / 304 0.33%
chr2:g.154854765G>T	Substitution	Stop Gained KCNJ3 E320*	1 / 304 0.33%
chr2:g.154854776G>T	Substitution	Missense KCNJ3 W323C	1 / 304 0.33%
chr2:g.154854943G>A	Substitution	Missense KCNJ3 S379N	1 / 304 0.33%
chr2:g.154854882A>T	Substitution	Stop Gained KCNJ3 K359*	1 / 304 0.33%
chr2:g.154699247G>A	Substitution	Missense KCNJ3 G158S	1 / 304 0.33%
chr2:g.154709759A>T	Substitution	Missense KCNJ3 S287C	1 / 304 0.33%
chr2:g.154699266T>A	Substitution	Missense KCNJ3 F164Y	1 / 304 0.33%
chr2:g.154709737C>T	Substitution	Synonymous KCNJ3 P279P	1 / 304 0.33%
chr2:g.154699327G>A	Substitution	Missense KCNJ3 M184I	1 / 304 0.33%
chr2:g.154699201C>T	Substitution	Synonymous KCNJ3 A142A	1 / 304 0.33%
chr2:g.154698930G>T	Substitution	Missense KCNJ3 R52L	1 / 304 0.33%

chr2:g.154699444G>T	Substitution	Missense KCNJ3 M223I	1 / 304<br>0.33%
chr2:g.154709650A>T	Substitution	Missense KCNJ3 E250D	1 / 304<br>0.33%
chr2:g.154698722G>A	Substitution	5 Prime UTR KCNJ3	1 / 304<br>0.33%
chr2:g.154699199G>A	Substitution	Missense KCNJ3 A142T	1 / 304<br>0.33%
chr2:g.154855099G>A	Substitution	Missense KCNJ3 G431E	1 / 304<br>0.33%
chr2:g.154698806G>A	Substitution	Missense KCNJ3 D11N	1 / 304<br>0.33%
chr2:g.154709748T>G	Substitution	Missense KCNJ3 L283R	1 / 304<br>0.33%
chr2:g.154699356T>A	Substitution	Missense KCNJ3 L194H	1 / 304<br>0.33%
chr2:g.154699045C>T	Substitution	Synonymous KCNJ3 T90T	1 / 304<br>0.33%
chr2:g.154699351G>A	Substitution	Synonymous KCNJ3 E192E	1 / 304<br>0.33%
chr2:g.154854865C>A	Substitution	Missense KCNJ3 T353N	1 / 304<br>0.33%
chr2:g.154699349G>A	Substitution	Missense KCNJ3 E192K	1 / 304<br>0.33%
chr2:g.154709733G>A	Substitution	Missense KCNJ3 S278N	1 / 304<br>0.33%
chr2:g.154855098G>A	Substitution	Missense KCNJ3 G431R	1 / 304<br>0.33%
chr2:g.154709705A>T	Substitution	Missense KCNJ3 T269S	1 / 304

				0.33%
☐	chr2:g.154699100C>T	Substitution	Missense KCNJ3 R109W	1 / 304 0.33%
☐	chr2:g.154698814G>A	Substitution	Synonymous KCNJ3 Q13Q	1 / 304 0.33%
☐	chr2:g.154699451G>A	Substitution	Missense KCNJ3 A226T	1 / 304 0.33%
☐	chr2:g.154855377G>A	Substitution	3 Prime UTR KCNJ3	1 / 304 0.33%
☐	chr2:g.154855022A>C	Substitution	Missense KCNJ3 K405N	1 / 304 0.33%
☐	chr2:g.154854837C>A	Substitution	Missense KCNJ3 Q344K	1 / 304 0.33%
☐	chr2:g.154855192C>T	Substitution	Missense KCNJ3 P462L	1 / 304 0.33%
☐	chr2:g.154699452C>T	Substitution	Missense KCNJ3 A226V	1 / 304 0.33%
☐	chr2:g.154855199C>T	Substitution	Synonymous KCNJ3 S464S	1 / 304 0.33%
☐	chr2:g.154698895G>T	Substitution	Missense KCNJ3 K40N	1 / 304 0.33%
☐	chr2:g.154855733G>C	Substitution	3 Prime UTR KCNJ3	1 / 304 0.33%
☐	chr2:g.154854953A>T	Substitution	Missense KCNJ3 R382S	1 / 304 0.33%
☐	chr2:g.154698875C>T	Substitution	Stop Gained KCNJ3 Q34*	1 / 304 0.33%
☐	chr2:g.154709662T>G	Substitution	Synonymous KCNJ3 G254G	1 / 304 0.33%

☐	chr2:g.154855180T>C	Substitution	Missense KCNJ3 M458T	1 / 304<br>0.33%
☐	chr2:g.154698888T>C	Substitution	Missense KCNJ3 V38A	1 / 304<br>0.33%
☐	chr2:g.154855961C>T	Substitution	3 Prime UTR KCNJ3	1 / 304<br>0.33%
☐	chr2:g.154709723G>T	Substitution	Missense KCNJ3 D275Y	1 / 304<br>0.33%
☐	chr2:g.154698902C>T	Substitution	Missense KCNJ3 R43W	1 / 304<br>0.33%
☐	chr2:g.154855654C>A	Substitution	3 Prime UTR KCNJ3	1 / 304<br>0.33%
☐	chr2:g.154698759G>T	Substitution	5 Prime UTR KCNJ3	1 / 304<br>0.33%
☐	chr2:g.154856079G>A	Substitution	3 Prime UTR KCNJ3	1 / 304<br>0.33%
☐	chr2:g.154709798G>C	Substitution	Missense KCNJ3 E300Q	1 / 304<br>0.33%
☐	chr2:g.154855132C>T	Substitution	Missense KCNJ3 S442L	1 / 304<br>0.33%
☐	chr2:g.154854958A>T	Substitution	Missense KCNJ3 N384I	1 / 304<br>0.33%
☐	chr2:g.154855038G>T	Substitution	Missense KCNJ3 D411Y	1 / 304<br>0.33%
☐	chr2:g.154699054G>T	Substitution	Synonymous KCNJ3 V93V	1 / 304<br>0.33%
☐	chr2:g.154709641C>T	Substitution	Synonymous KCNJ3 D247D	1 / 304<br>0.33%
☐	chr2:g.154698814G>T	Substitution	Missense KCNJ3 Q13H	1 / 304

			0.33%
chr2:g.154699207C>A	Substitution	Synonymous KCNJ3 I144I	1 / 304 0.33%
chr2:g.154699084G>T	Substitution	Missense KCNJ3 W103C	1 / 304 0.33%
chr2:g.154698633A>T	Substitution	5 Prime UTR KCNJ3	1 / 304 0.33%
chr2:g.154854969T>A	Substitution	Missense KCNJ3 C388S	1 / 304 0.33%
chr2:g.154698948G>T	Substitution	Missense KCNJ3 G58V	1 / 304 0.33%
chr2:g.154854814delGATTCTTT...	Deletion	Frameshift KCNJ3 F337Lfs*9	1 / 304 0.33%
chr2:g.154699440A>G	Substitution	Missense KCNJ3 H222R	1 / 304 0.33%
chr2:g.154855277_154855278insA	Insertion	Frameshift KCNJ3 L492Ifs*7	1 / 304 0.33%
chr2:g.154855904A>G	Substitution	3 Prime UTR KCNJ3	1 / 304 0.33%
chr2:g.154855043T>C	Substitution	Synonymous KCNJ3 F412F	1 / 304 0.33%
chr2:g.154855698A>G	Substitution	3 Prime UTR KCNJ3	1 / 304 0.33%
chr2:g.154855269C>A	Substitution	Missense KCNJ3 L488I	1 / 304 0.33%
chr2:g.154698758G>T	Substitution	5 Prime UTR KCNJ3	1 / 304 0.33%
chr2:g.154855495G>A	Substitution	3 Prime UTR KCNJ3	1 / 304 0.33%

☐	chr2:g.154709748T>C	Substitution	Missense KCNJ3 L283P	1 / 304<br>0.33%
☐	chr2:g.154698895G>C	Substitution	Missense KCNJ3 K40N	1 / 304<br>0.33%
☐	chr2:g.154709712G>A	Substitution	Missense KCNJ3 C271Y	1 / 304<br>0.33%
☐	chr2:g.154698829G>A	Substitution	Synonymous KCNJ3 S18S	1 / 304<br>0.33%
☐	chr2:g.154699124G>T	Substitution	Missense KCNJ3 V117F	1 / 304<br>0.33%
☐	chr2:g.154709673G>A	Substitution	Missense KCNJ3 G258E	1 / 304<br>0.33%
☐	chr2:g.154854961C>A	Substitution	Missense KCNJ3 S385Y	1 / 304<br>0.33%
☐	chr2:g.154698948G>A	Substitution	Missense KCNJ3 G58D	1 / 304<br>0.33%
☐	chr2:g.154699183C>A	Substitution	Missense KCNJ3 F136L	1 / 304<br>0.33%
☐	chr2:g.154855128A>C	Substitution	Missense KCNJ3 S441R	1 / 304<br>0.33%
☐	chr2:g.154855095T>G	Substitution	Missense KCNJ3 L430V	1 / 304<br>0.33%
☐	chr2:g.154699306C>T	Substitution	Synonymous KCNJ3 I177I	1 / 304<br>0.33%
☐	chr2:g.154699343C>T	Substitution	Missense KCNJ3 R190C	1 / 304<br>0.33%
☐	chr2:g.154699209G>C	Substitution	Missense KCNJ3 G145A	1 / 304<br>0.33%
☐	chr2:g.154698983G>A	Substitution	Missense KCNJ3 D70N	1 / 304

				0.33%
	chr2:g.154698745C>T	Substitution	5 Prime UTR KCNJ3	1 / 304<br>0.33%
	chr2:g.154709761C>A	Substitution	Missense KCNJ3 S287R	1 / 304<br>0.33%
	chr2:g.154854860C>T	Substitution	Synonymous KCNJ3 V351V	1 / 304<br>0.33%
	chr2:g.154698982G>A	Substitution	Synonymous KCNJ3 S69S	1 / 304<br>0.33%
	chr2:g.154856445T>G	Substitution	3 Prime UTR KCNJ3	1 / 304<br>0.33%
	chr2:g.154698845T>C	Substitution	Synonymous KCNJ3 L24L	1 / 304<br>0.33%
	chr2:g.154854793C>T	Substitution	Missense KCNJ3 P329L	1 / 304<br>0.33%
	chr2:g.154856363T>G	Substitution	3 Prime UTR KCNJ3	1 / 304<br>0.33%
	chr2:g.154855213A>C	Substitution	Missense KCNJ3 D469A	1 / 304<br>0.33%
	chr2:g.154699051C>T	Substitution	Synonymous KCNJ3 T92T	1 / 304<br>0.33%
	chr2:g.154854777G>T	Substitution	Missense KCNJ3 G324C	1 / 304<br>0.33%
	chr2:g.154699190G>A	Substitution	Missense KCNJ3 E139K	1 / 304<br>0.33%
	chr2:g.154855943C>A	Substitution	3 Prime UTR KCNJ3	1 / 304<br>0.33%
	chr2:g.154698946C>T	Substitution	Synonymous KCNJ3 H57H	1 / 304<br>0.33%

☐	chr2:g.154699395G>T	Substitution	Missense KCNJ3 G207V	1 / 304<br>0.33%
☐	chr2:g.154698988C>T	Substitution	Synonymous KCNJ3 L71L	1 / 304<br>0.33%
☐	chr2:g.154699330C>A	Substitution	Synonymous KCNJ3 S185S	1 / 304<br>0.33%
☐	chr2:g.154856005G>A	Substitution	3 Prime UTR KCNJ3	1 / 304<br>0.33%
☐	chr2:g.154854883A>T	Substitution	Missense KCNJ3 K359I	1 / 304<br>0.33%
☐	chr2:g.154856383delT	Deletion	3 Prime UTR KCNJ3	1 / 304<br>0.33%
☐	chr2:g.154855184A>T	Substitution	Missense KCNJ3 L459F	1 / 304<br>0.33%
☐	chr2:g.154855318G>T	Substitution	3 Prime UTR KCNJ3	1 / 304<br>0.33%
☐	chr2:g.154855251G>A	Substitution	Missense KCNJ3 A482T	1 / 304<br>0.33%
☐	chr2:g.154855096T>C	Substitution	Missense KCNJ3 L430S	1 / 304<br>0.33%
☐	chr2:g.154699359T>A	Substitution	Missense KCNJ3 M195K	1 / 304<br>0.33%
☐	chr2:g.154855689C>T	Substitution	3 Prime UTR KCNJ3	1 / 304<br>0.33%
☐	chr2:g.154854733C>G	Substitution	Missense KCNJ3 T309S	1 / 304<br>0.33%
☐	chr2:g.154698981C>G	Substitution	Missense KCNJ3 S69W	1 / 304<br>0.33%
☐	chr2:g.154698705T>A	Substitution	5 Prime UTR KCNJ3	1 / 304

			0.33%
chr2:g.154855066G>A	Substitution	Missense KCNJ3 S420N	1 / 304 0.33%
chr2:g.154699137C>T	Substitution	Missense KCNJ3 T121M	1 / 304 0.33%
chr2:g.154709699C>A	Substitution	Missense KCNJ3 P267T	1 / 304 0.33%
chr2:g.154854782T>C	Substitution	Synonymous KCNJ3 H325H	1 / 304 0.33%
chr2:g.154699005A>G	Substitution	Missense KCNJ3 D77G	1 / 304 0.33%
chr2:g.154698825C>T	Substitution	Missense KCNJ3 T17I	1 / 304 0.33%
chr2:g.154855246G>T	Substitution	Missense KCNJ3 G480V	1 / 304 0.33%
chr2:g.154855247A>T	Substitution	Synonymous KCNJ3 G480G	1 / 304 0.33%
chr2:g.154709727C>T	Substitution	Missense KCNJ3 A276V	1 / 304 0.33%
chr2:g.154699170C>A	Substitution	Missense KCNJ3 S132Y	1 / 304 0.33%
chr2:g.154698780C>T	Substitution	Missense KCNJ3 S2F	1 / 304 0.33%
chr2:g.154855307C>T	Substitution	Synonymous KCNJ3 F500F	1 / 304 0.33%
chr2:g.154855081delA	Deletion	Frameshift KCNJ3 A427Pfs*9	1 / 304 0.33%
chr2:g.154698694C>G	Substitution	5 Prime UTR KCNJ3	1 / 304 0.33%

☐	chr2:g.154709620G>A	Substitution	Synonymous KCNJ3 E240E	1 / 304<br>0.33%
☐	chr2:g.154699295G>A	Substitution	Missense KCNJ3 A174T	1 / 304<br>0.33%
☐	chr2:g.154709681C>T	Substitution	Stop Gained KCNJ3 Q261*	1 / 304<br>0.33%
☐	chr2:g.154699060G>A	Substitution	Stop Gained KCNJ3 W95*	1 / 304<br>0.33%
☐	chr2:g.154699456G>T	Substitution	Missense KCNJ3 Q227H	1 / 304<br>0.33%
☐	chr2:g.154709633C>A	Substitution	Missense KCNJ3 P245T	1 / 304<br>0.33%
☐	chr2:g.154699051C>A	Substitution	Synonymous KCNJ3 T92T	1 / 304<br>0.33%
☐	chr2:g.154709813A>G	Substitution	Missense KCNJ3 T305A	1 / 304<br>0.33%
☐	chr2:g.154856005G>T	Substitution	3 Prime UTR KCNJ3	1 / 304<br>0.33%
☐	chr2:g.154709820G>C	Substitution	Splice Donor KCNJ3 X307_splice	1 / 304<br>0.33%
☐	chr2:g.154699402C>A	Substitution	Synonymous KCNJ3 L209L	1 / 304<br>0.33%
☐	chr2:g.154854995T>C	Substitution	Synonymous KCNJ3 T396T	1 / 304<br>0.33%
☐	chr2:g.154709696_154709697insC	Insertion	Frameshift KCNJ3 L268Pfs*15	1 / 304<br>0.33%
☐	chr2:g.154698857G>T	Substitution	Missense KCNJ3 G28W	1 / 304<br>0.33%
☐	chr2:g.154855739G>T	Substitution	3 Prime UTR KCNJ3	1 / 304

0.33%

☐	chr2:g.154699430C>T	Substitution	Missense KCNJ3 R219C	1 / 304 0.33%
☐	chr2:g.154854984G>T	Substitution	Missense KCNJ3 D393Y	1 / 304 0.33%
☐	chr2:g.154699365G>A	Substitution	Missense KCNJ3 S197N	1 / 304 0.33%
☐	chr2:g.154698927G>A	Substitution	Missense KCNJ3 G51D	1 / 304 0.33%
☐	chr2:g.154698788C>T	Substitution	Stop Gained KCNJ3 R5*	1 / 304 0.33%
☐	chr2:g.154699228C>T	Substitution	Synonymous KCNJ3 I151I	1 / 304 0.33%
