# Supplementary material for: Consequences of somatic mutations of GIRK1 detected in primary malign tumors on expression and function of G-protein activated, inwardly rectifying, K+ channels
Source: Front Oncol. 2022 Oct 31;12:998907. doi: 10.3389/fonc.2022.998907 (PMC9724741; doi:10.3389/fonc.2022.998907)
Supplement: Supplementary file 2 [file DataSheet_2.pdf]

Supplementary Table 2.:

	ΔF mean	SEM	N	p-value (vs. natives)	p-value (mutant vs. WT)	p-value (-G4 vs. +G4)
native	0,01	0,00	109			
WT	0,28	0,03	136	<0,001		<0,001
WT+G4	1,00	0,04	142	<0,001		
S132Y	0,21	0,03	25	<0,001	0,468	<0,001
S132Y+G4	0,66	0,08	24	<0,001	0,007	
L135I	0,20	0,03	25	<0,001	0,135	<0,001
L135I+G4	0,77	0,12	23	<0,001	0,729	
F136L	0,28	0,06	19	0,060	>0,999	<0,001
F136L+G4	0,65	0,11	20	0,015	0,007	
E139K	0,26	0,03	19	<0,001	>0,999	<0,001
E139K+G4	1,00	0,13	19	<0,001	>0,999	
E140M	0,11	0,02	14	0,045	<0,001	<0,001
E140M+G4	1,00	0,12	14	<0,001	0,830	
A142T	0,43	0,08	23	0,060	0,748	<0,001
A142T+G4	2,48	0,35	19	<0,001	0,055	
G145A	0,32	0,08	23	0,072	>0,999	0,015
G145A+G4	0,66	0,13	24	0,045	0,570	
R149Q	0,23	0,02	24	<0,001	0,748	<0,001
R149Q+G4	1,36	0,33	24	0,101	>0,999	
R149P	0,36	0,08	24	0,052	>0,999	<0,001
R149P+G4	1,12	0,24	23	0,060	>0,999	
I151N	0,13	0,02	28	0,008	<0,001	0,503
I151N+G4	0,29	0,09	29	0,015	<0,001	
G158S	0,11	0,02	27	<0,001	<0,001	0,393
G158S+G4	0,15	0,06	29	0,262	<0,001	
G178D	0,14	0,02	24	0,015	<0,001	<0,001
G178D+G4	1,28	0,18	24	<0,001	0,830	
M184I	0,23	0,03	20	0,024	0,748	0,004
M184I+G4	0,99	0,10	17	0,101	>0,999	
S185Y	0,17	0,03	23	0,024	0,072	<0,001
S185Y+G4	1,23	0,13	24	<0,001	0,830	
Q186R	0,29	0,04	22	<0,001	>0,999	<0,001
Q186R+G4	1,67	0,24	19	<0,001	0,729	

Supplementary Table 3.:

	I_{total} mean	SEM	N	p-value (vs. natives)	p-value (mutant vs. WT)	p-value (-G4 vs. +G4)
native	0,02	0,00	109			
G4 (homo)	0,20	0,02	130	<0,001		
WT	0,07	0,00	133	<0,001		<0,001
WT+G4	1,00	0,03	137	<0,001		
S132Y	0,03	0,01	25	0,635	<0,001	<0,001
S132Y+G4	0,57	0,07	24	0,004	<0,001	
L135I	0,04	0,00	25	0,012	<0,001	<0,001
L135I+G4	0,95	0,10	23	<0,001	0,649	
F136L	0,02	0,00	24	>0,999	<0,001	<0,001
F136L+G4	0,72	0,07	25	<0,001	<0,001	
E139K	0,02	0,01	25	>0,999	<0,001	<0,001
E139K+G4	0,21	0,04	24	>0,999	<0,001	
E140M	0,03	0,00	24	>0,999	<0,001	<0,001
E140M+G4	1,22	0,13	23	<0,001	0,252	
A142T	0,10	0,01	20	<0,001	0,034	<0,001
A142T+G4	1,31	0,14	20	<0,001	0,166	
G145A	0,03	0,00	24	>0,999	<0,001	<0,001
G145A+G4	0,32	0,09	23	0,678	<0,001	
R149Q	0,02	0,00	24	>0,999	<0,001	<0,001
R149Q+G4	0,71	0,09	25	<0,001	0,039	
R149P	0,02	0,00	24	>0,999	<0,001	<0,001
R149P+G4	0,72	0,12	23	0,018	0,166	
I151N	0,02	0,00	23	>0,999	<0,001	<0,001
I151N+G4	0,42	0,09	24	0,480	<0,001	
G158S	0,02	0,00	27	>0,999	<0,001	<0,001
G158S+G4	0,21	0,03	29	>0,999	<0,001	
G178D	0,04	0,01	25	0,535	<0,001	<0,001
G178D+G4	0,68	0,06	25	<0,001	<0,001	
M184I	0,13	0,01	16	<0,001	<0,001	<0,001
M184I+G4	1,63	0,22	15	<0,001	0,093	
S185Y	0,03	0,00	23	0,479	<0,001	<0,001
S185Y+G4	0,27	0,03	24	0,480	<0,001	
Q186R	0,05	0,00	24	<0,001	<0,001	<0,001
Q186R+G4	0,43	0,07	23	0,039	<0,001	

Supplementary Table 4.:

	I_{ACh}/I_{total} mean	*SEM*	*N*	*p-value (mutant vs. WT)*
WT	0,42	0,01	137	
S132Y	0,42	0,03	24	>0,999
L135I	0,37	0,03	23	0,980
F136L	0,42	0,03	25	>0,999
E139K	0,43	0,03	24	>0,999
E140M	0,31	0,04	23	0,143
A142T	0,42	0,03	20	>0,999
G145A	0,34	0,03	23	0,234
R149Q	0,43	0,04	25	>0,999
R149P	0,38	0,04	23	>0,999
I151N	0,51	0,02	24	*0,049*
G158S	0,49	0,03	29	0,237
G178D	0,37	0,02	25	>0,999
M184I	0,46	0,03	15	>0,999
S185Y	0,28	0,02	24	*<0,001*
Q186R	0,44	0,03	23	>0,999
