# Supplementary material for: Consequences of somatic mutations of GIRK1 detected in primary malign tumors on expression and function of G-protein activated, inwardly rectifying, K+ channels
Source: Front Oncol. 2022 Oct 31;12:998907. doi: 10.3389/fonc.2022.998907 (PMC9724741; doi:10.3389/fonc.2022.998907)
Supplement: Supplementary file 3 [file DataSheet_3.pdf]

SUPPLEMENTARY FIGURES:

Consequences of somatic mutations of GIRK1 detected in primary malign tumors on expression and function of G-protein activated, inwardly rectifying, K^+ channels

Brigitte PELZMANN[1,2*], Ahmed HATAB[1,2], Susanne SCHERUEBEL[1,2], Sonja LANGTHALER[3], Theresa RIENMUELLER[3], Armin SOKOLOWSKI[4], Astrid GORISCHEK[1,2], Dieter PLATZER[1,2], Klaus ZORN-PAULY[1,2], Stephan W. JAHN[5], Thomas BAUERNHOFER[6,2] and Wolfgang SCHREIBMAYER[1,2].

[1]: Gottfried Schatz Research Center for Cell Signaling, Metabolism and Aging, Medical Physics and Biophysics, Medical University of Graz, Graz, Austria.

[2]: Research Unit on Ion Channels and Cancer Biology, Medical University of Graz, Graz, Austria.

[3]: Institute of Health Care Engineering with European Testing Center of Medical Devices, Graz University of Technology, Graz, Austria.

[4]: Department of Dental Medicine and Oral Health, Medical University of Graz, Graz, Austria.

[5]: Diagnostic & Research Institute of Pathology, Medical University of Graz, Graz, Austria.

[6]: Division of Oncology, Department of Internal Medicine, Medical University of Graz, Graz, Austria.

[*]: corresponding author: Gottfried Schatz Research Center for Cell Signaling, Metabolism and Aging, Research Unit "Ion Channels and Cancer Biology", Medical Physics and Biophysics, Medical University of Graz, Graz, Austria.

Supplementary Figure 1: Somatic KCNJ3 mutations detected in different types of cancer

A

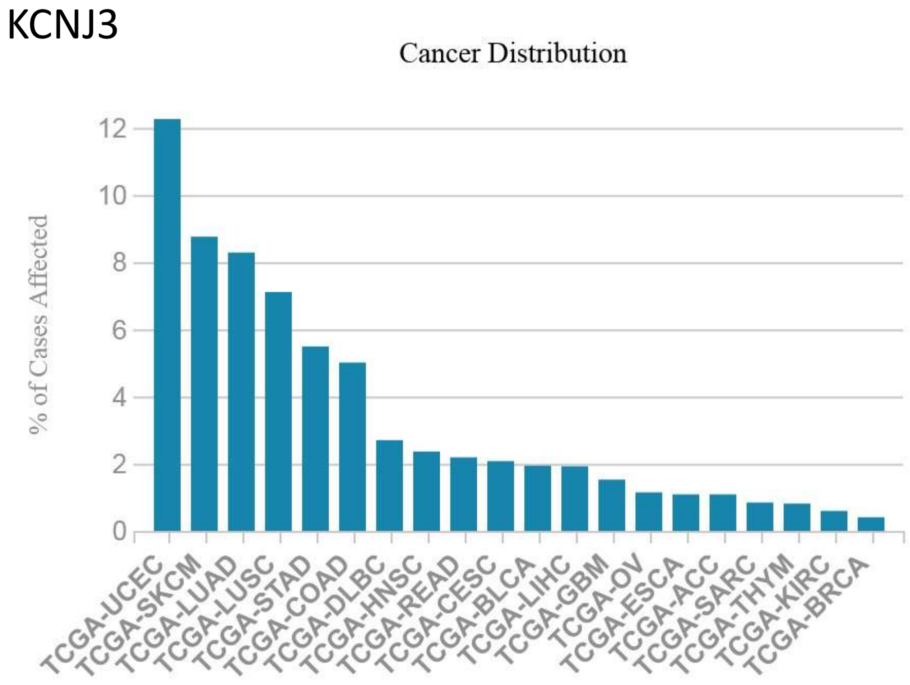

B

Project	*Disease Type*	*Site*	*# Affected Cases*	*# Mutations*
TCGA-UCEC	Uterine Corpus Endometrial Carcinoma	Uterus	65 / 530 (12.26%)	112
TCGA-SKCM	Skin Cutaneous Melanoma	Skin	41 / 468 (8.76%)	48
TCGA-LUAD	Lung Adenocarcinoma	Lung	47 / 567 (8.29%)	51
TCGA-LUSC	Lung Squamous Cell Carcinoma	Lung	35 / 492 (7.11%)	37
TCGA-STAD	Stomach Adenocarcinoma	Stomach	24 / 437 (5.49%)	27
TCGA-COAD	Colon Adenocarcinoma	Colorectal	20 / 399 (5.01%)	23
TCGA-DLBC	Lymphoid Neoplasm Diffuse Large B-cell Lymphoma	Lymph Nodes	1 / 37 (2.70%)	1
TCGA-HNSC	Head and Neck Squamous Cell Carcinoma	Head and Neck	12 / 508 (2.36%)	13
TCGA-READ	Rectum Adenocarcinoma	Colorectal	3 / 137 (2.19%)	3
TCGA-CESC	Cervical Squamous Cell Carcinoma and Endocervical Adenocarcinoma	Cervix	6 / 289 (2.08%)	9

Supplementary Figure 1A: Percentage of primary tumors of an experimental cohort affected by somatic mutations in KCNJ3 as revealed by the GDC Data Portal (https://portal.gdc.cancer.gov). Tumor type is stated below the horizontal axis.

Supplementary Figure 1B: Table showing details of the ten experimental cohorts that were most affected by somatic mutations in KCNJ3.

Supplementary Figure 2: Somatic mutations in the GIRK1 subunit:

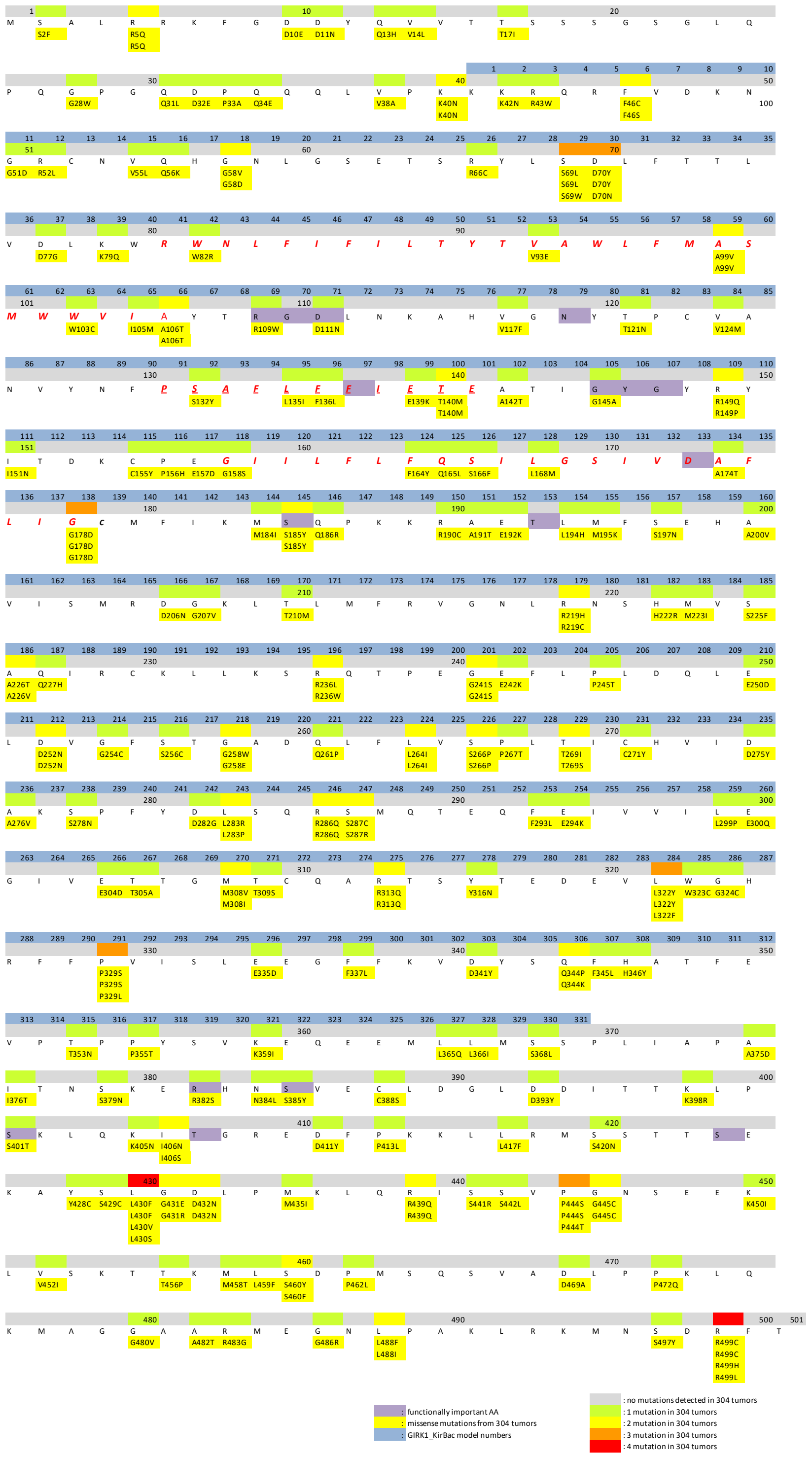

Amino acid numbering according to entire full length variant 1a. Regions comprising the pore helix and the transmembrane helix 2 are emphasized in red. Somatic mutations detected are highlighted in *green* (1 mutation found), *yellow* (2 mutations found), *orange* (3 mutations found) and *red* (4 mutations found out of 304 cases). Multiple appearance of a mutation underneath an amino acid residue indicates multiple occurence in different tumors of the sample cohort. Numbers in blue boxes: Aminoacid numbering of KirBac structure according to [1].

Supplementary Figure 3: GIRK1^{I151N} and GIRK1^{G158S} tested for the ability to form functional homotetrameric ion channels.

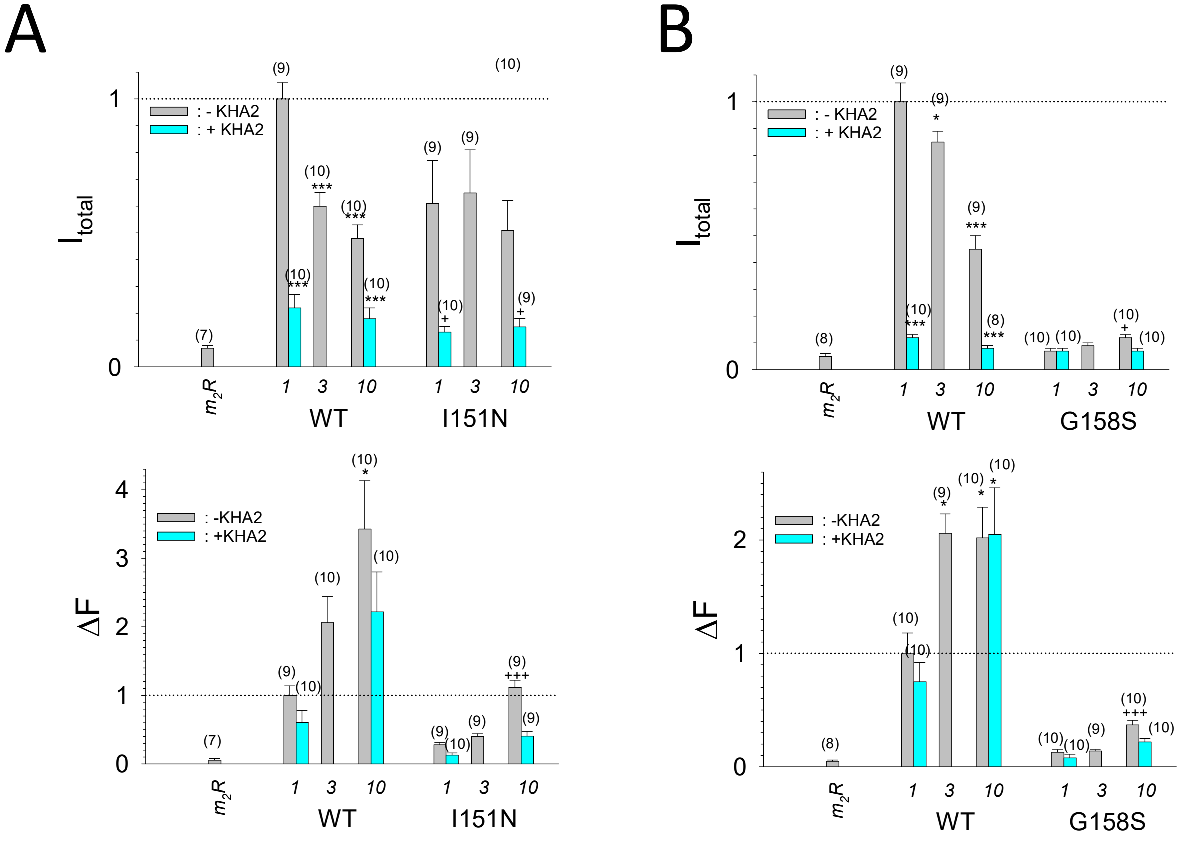

Supplementary *Figure 3A:* I_{total} (*upper*) and ΔF values (*lower*), resulting from injection of different amounts of mRNA (in ng at bottom of graph). GIRK1^{WT} was compared to GIRK1^{I151N}. Bars represent mean values of the given experimental group. *Grey:* without coinjection of KHA2 antisense oligonucleotide, *cyan:* KHA2 antisense oligonucleotide was coinjected with mRNA. Number of oocytes given in parenthesis above the bars. Whiskers denote SEM. *: the mean value differs statistically significant from the control (GIRK1^{WT} or GIRK1^{I151N}, respectively; 1ng mRNA without KHA2 antisense oligonucleotide coinjected) at the $p>0.05$ level.

Supplementary *Figure 3B:* Similar to *3A*, but G158S was tested for functional GIRK1 homotetramer formation.

Supplementary Figure 4: Ratio of I_{ACh}/I_{total} for the different somatic mutations of the GIRK1 subunit, when coexpressed with GIRK4.

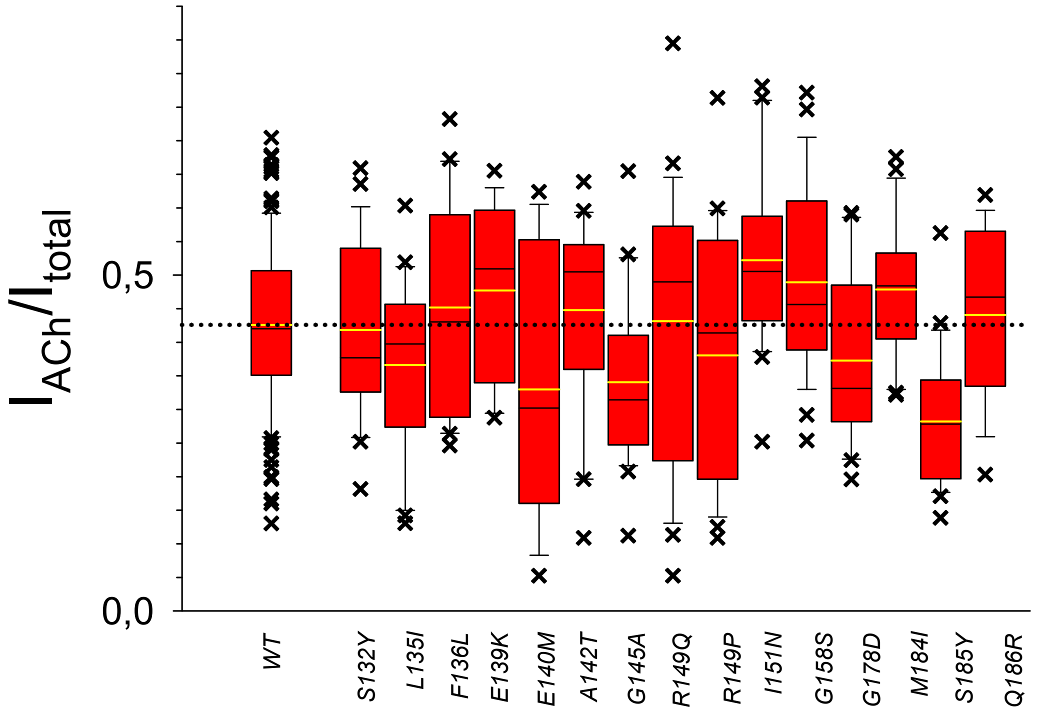

I_{ACh}/I_{total} ratios of different GIRK1^{eGFP} subunit mutations, relative to WT. GIRK1 was coinjected with GIRK4 mRNA. Box plots comprise 25% and 75% percentiles. Median (solid black line within box) and average value (yellow line) indicated within box. Whiskers denote 10% and 90% percentiles. Oocytes having values above 90% and below 10% percentiles are shown as cross. See supplementary Table 4 for exact values, numbers and statistics. *Dotted line* indicates the mean value of WT/GIRK4.

Reference:

1. Nishida M, Cadene M, Chait BT, MacKinnon R: Crystal structure of a Kir3.1-prokaryotic Kir channel chimera. *The EMBO journal* 2007, 26(17):4005-4015.
